# A Genome-Wide Association Study Identifies Variants Underlying the *Arabidopsis thaliana* Shade Avoidance Response

**DOI:** 10.1371/journal.pgen.1002589

**Published:** 2012-03-15

**Authors:** Daniele L. Filiault, Julin N. Maloof

**Affiliations:** Department of Plant Biology, University of California Davis, Davis, California, United States of America; University of Georgia, United States of America

## Abstract

Shade avoidance is an ecologically and molecularly well-understood set of plant developmental responses that occur when the ratio of red to far-red light (R∶FR) is reduced as a result of foliar shade. Here, a genome-wide association study (GWAS) in *Arabidopsis thaliana* was used to identify variants underlying one of these responses: increased hypocotyl elongation. Four hypocotyl phenotypes were included in the study, including height in high R∶FR conditions (simulated sun), height in low R∶FR conditions (simulated shade), and two different indices of the response of height to low R∶FR. GWAS results showed that variation in these traits is controlled by many loci of small to moderate effect. A known *PHYC* variant contributing to hypocotyl height variation was identified and lists of significantly associated genes were enriched in *a priori* candidates, suggesting that this GWAS was capable of generating meaningful results. Using metadata such as expression data, GO terms, and other annotation, we were also able to identify variants in candidate *de novo* genes. Patterns of significance among our four phenotypes allowed us to categorize associations into three groups: those that affected hypocotyl height without influencing shade avoidance, those that affected shade avoidance in a height-dependent fashion, and those that exerted specific control over shade avoidance. This grouping allowed for the development of explicit hypotheses about the genetics underlying shade avoidance variation. Additionally, the response to shade did not exhibit any marked geographic distribution, suggesting that variation in low R∶FR–induced hypocotyl elongation may represent a response to local conditions.

## Author Summary

The goal of this work was to identify genetic variants underlying a well-characterized environmental response, the elongation of *Arabidopsis thaliana* hypocotyls (seedling stems) in response to shade, otherwise known as shade avoidance. We performed a genome-wide association study with four phenotypes: absolute hypocotyl height of plants grown in both simulated sun and shade and two measures of how height responded to shade. With this study, we confirmed previous findings that variants in two photoreceptors were associated with hypocotyl height variation. We also found associations with genetic variants in previously-identified shade avoidance genes, as well as with variants in genes not typically considered part of the shade avoidance pathway. By examining patterns of which of the four phenotypes were associated with each gene, we were then able to discriminate between genetic variants that have a general role in hypocotyl height variation and variants that are specifically involved in the shade avoidance response. We also found that shade avoidance was not broadly associated with geography, suggesting that variation in this trait may be due to local differences in light quality.

## Introduction

Since plants are sessile organisms that rely on the harvest of light to fulfill their energy requirements, the ability to monitor the ambient light environment has been key to their evolutionary success. Faced with the challenge of modulating their development to best suit changing light environments, plants have evolved a sophisticated array of photoreceptors to comprehensively survey both light quality and quantity [1]. These photoreceptors are integrated into key developmental pathways, allowing for efficient optimization of development. One well-studied case in which changes in light quality elicit specific developmental responses is shade avoidance [2].

When sunlight is intercepted by a plant canopy, plant pigments absorb light in the red and blue portions of the spectrum to use as energy for photosynthesis, while far-red light passes through the canopy relatively unimpeded. As a result, the ratio of R∶FR light is reduced in canopy shade or when neighboring plants are present. Plants sense this change in light quality primarily through type II (light stable) phytochromes and initiate a suite of plastic developmental responses known as shade avoidance [2]. These responses include elongation of plant organs, including hypocotyls, internodes, and petioles, increased leaf angle, and acceleration of flowering. In natural plant communities, the shade avoidance response has long been the subject of ecological genetic studies. Initially, researchers observed that the phytochrome-mediated response to low R∶FR in species originating from shaded environments was lower than that of species from open environments [3]. Dudley and Schmitt [4] observed a similar pattern between populations of a single species, *Impatiens capensis*. A subsequent physiological manipulation study of this species confirmed that in natural populations, shade avoidance elongation responses are indeed an example of adaptive plasticity [4], while a genetic manipulation study of transgenic tobacco and *Brassica* demonstrated that this adaptive plasticity could be phytochrome-mediated [5]. Interestingly, R∶FR-mediated shade avoidance elongation has also been shown to be adaptive in the model plant *Arabidopsis thaliana* [6], and in a survey of 105 *Arabidopsis* accessions, Botto and Smith observed considerable natural variation in hypocotyl elongation in response to low R∶FR [7]. Therefore, evolutionary and ecological genetics studies of shade avoidance present an opportunity to use the extensive genetic resources of *Arabidopsis* to investigate an adaptive trait.

Shade avoidance is also relevant to agricultural settings, since high planting densities can create low R∶FR conditions, triggering shade avoidance and thereby decreasing yield [8]. As a result, extensive studies of the molecular nature of shade avoidance have been undertaken, particularly in *Arabidopsis*. In this species, light stable phytochromes, especially phytochrome B, initiate shade avoidance [2]. In response to a reduction in R∶FR, these proteins undergo a conformational change to the inactive (Pr) state. Through mechanisms that are as yet unclear, but that most likely involve interactions with the transcription factors PIF4 and PIF5 [9], this conformational change triggers the upregulation of a suite of transcription factors, including *HFR1*, *ATHB-2*, *ATHB-4*, *PIL1*, *PAR1*, and *PAR2* [10]–[12]. This upregulation ultimately leads to hypocotyl elongation through increased synthesis and modulated signaling of plant hormones including auxin, gibberellic acid, brassinosteroids, cytokinins, and ethylene [13]. Of these hormones, the involvement of auxin and gibberellic acid (GA) is best supported. Genes controlling both auxin synthesis (*TAA1* and *YUCCAs*) [14], [15] as well as auxin transport (*BIG*, *PIN3*) [16], [17] have been shown to play roles in low R∶FR-mediated elongation. Additionally, genes encoding two auxin-responsive proteins, IAA19 and IAA29, are upregulated in response to shade [10], [18]. The importance of GA signaling in shade avoidance is evidenced not only by the low R∶FR-induced upregulation of two gibberellic acid (GA) synthesis genes, *GA20ox1* and *GA20ox2* [19], but also by the role of the DELLA proteins. These negative regulators of PIF activity are degraded as a result of increased GA synthesis under low R∶FR conditions and therefore serve as integrators of light and hormone signaling [20]. Although a completely unified understanding of the shade avoidance pathway remains elusive, the reasonably well-understood molecular nature of the shade avoidance response is another reason why this phenotype is well-suited for studies of natural variation that seek to uncover the genetic control of adaptive traits.

In fact, quantitative genetics studies of *Arabidopsis* natural variation have been successful in identifying genetic variation in the phytochrome B-mediated signaling pathway. QTL studies have identified natural variants of *PHYB* and *ELF3* that impart a difference in light sensitivity [21]–[23], while researchers taking a candidate gene approach have identified alleles of both *PHYD* and *PIF4* that contribute to shade avoidance variation [24], [25]. These studies, however, have been somewhat limited in scope, as QTL analyses with recombinant inbred lines can only assess the variation present between the parental accessions, while candidate gene approaches rely entirely on previous knowledge about the pathway in question. Studying shade avoidance responses using a genome-wide association study (GWAS), therefore, expands upon this work in two ways. First, by examining genetic variation in many accessions simultaneously, GWAS not only tests more genetic variation than the QTL approach, it also emphasizes variation that is more likely to be broadly important in natural populations. Secondly, the use of high-density genome-wide SNPs in GWAS not only allows for truly *de novo* candidate gene discovery, but also enables a comprehensive view of genetic architecture of the traits in question. The goal of this study was to capitalize upon these strengths of GWAS, combined with the strategy of representing the shade avoidance response as a genotype by environment (GxE) interaction, to identify genetic variants underlying natural variation in shade avoidance.

## Results/Discussion

### Measurement of Natural Variation

**Figure 1:**
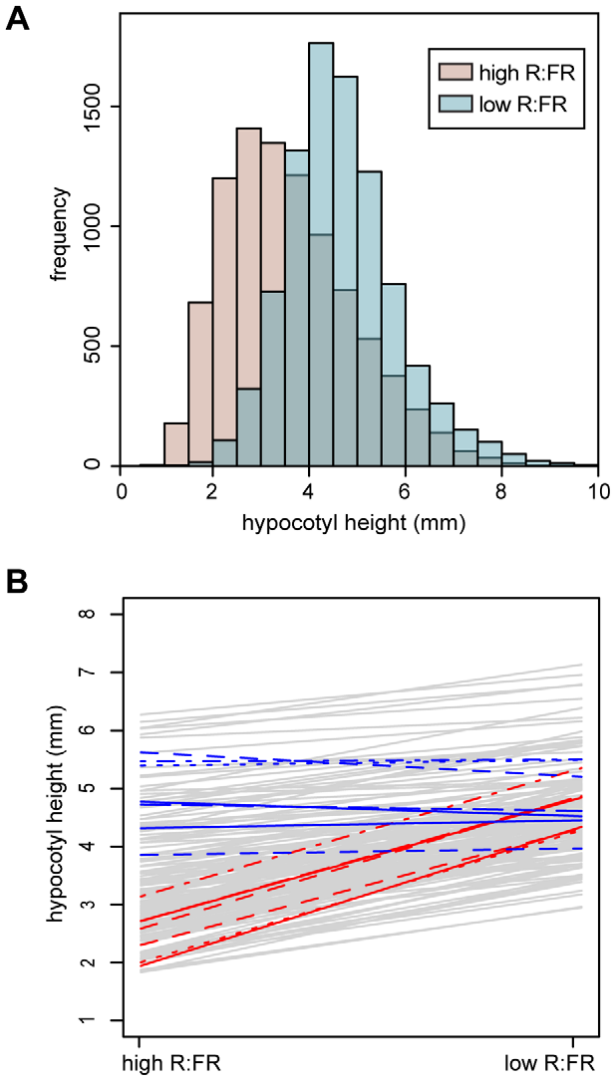
Hypocotyl height phenotypes. (A) Histograms of hypocotyl height for seedings grown under high R∶FR (pink) or low R∶FR (blue) treatments. (B) Hypocotyl height reaction norms of 180 *Arabidopsis* accessions. Reaction norms for the seven highest-responding accessions (in descending order: 9057, 8242, 6929, 6009, 6914, 6968, 8231) are plotted with red lines, while reaction norms for the seven lowest-responding accession (in ascending order: 6928, 8304, 7515, 6943, 8395, 6916, 8337) are plotted in blue. The three lowest-responding accessions showed a slight negative response to low R∶FR (−0.41, −0.25, and −0.11 millimeters).

To assess the extent of natural variation in the hypocotyl response to shade, 180 *Arabidopsis thaliana* accessions (Table S1) were grown in both simulated sun (high R∶FR) and simulated shade (low R∶FR) conditions. This set of accessions not only included samples covering the broad range of *Arabidopsis* throughout the world, but also incorporated focused subsampling from Sweden to improve our ability to detect local adaptation. As expected, when all accessions were considered together, hypocotyls of seedings grown in low R∶FR were taller than those of seedlings grown in high R∶FR (*t*-test *P*-value
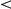
2.2e-16) (Figure 1A).

Next we asked if phenotypic variation among the accessions could be due to genetic variation. Significant differences in hypocotyl height among the accessions were observed in both light conditions (*P*-value
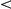
2.2e-16). Broad-sense heritability of hypocotyl height was 0.54 for the high R∶FR- and 0.44 for the low R∶FR-treated seedlings. Variation in the shade avoidance response among the accessions was assessed by fitting a mixed linear model that included genetic (accession), environment (light treatment), and GxE (accession×light) components. Because the experiment was repeated with chamber-swapping, we also included an experiment effect in the model. All variables in the model were significant (*P*-value
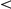
2.2e-16, Table 1), indicating significant differences in the shade avoidance response among accessions. Similarly, the genetic correlation between environments was 0.78, revealing that at least part of the genetic control of hypocotyl height varied between the two environments [26], [27]. The fitted values for hypocotyl height in high R∶FR, hypocotyl height in low R∶FR, and response to low R∶FR for each genotype were extracted from the full mixed-effects model for subsequent GWAS analysis (Figure S1).

**Table 1:** Parameters from the phenotype mixed effects model.

Effect	Variance	Standard Deviation
Genotype	1.01	1.00
Genotype×Environment	0.32	0.56
Experiment	0.04	0.20
Residual	0.72	0.85

Estimates of the variance and standard deviation of random effects from the mixed effect model used to generate GWAS phenotypes.

**Figure 2:**
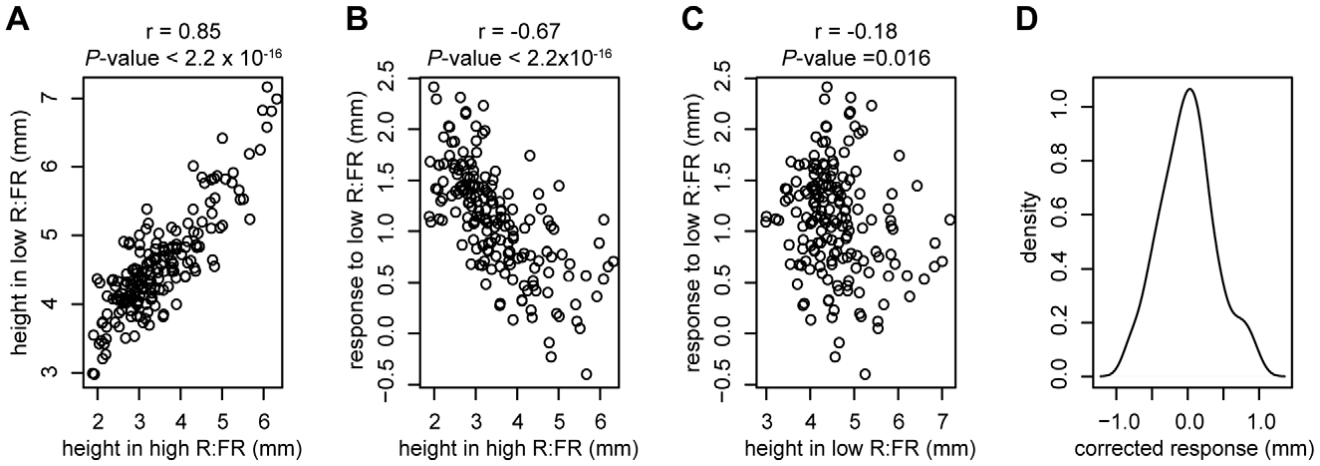
Relationships between phenotypes. Correlations between height in high R∶FR and height in low R∶FR (A), height in high R∶FR and response to low R∶FR (B), and height in low R∶FR and response to low R∶FR (C). The kernel density plot in panel D shows the distribution of the corrected response phenotype (residuals from a regression of response to low R∶FR against height in high R∶FR).

To explore patterns in variation among these phenotypes, a reaction norm plot was generated from the modeled phenotypic values (Figure 1B). The accessions that were most responsive to low R∶FR tended to have shorter hypocotyls in high R∶FR, while the least-responsive accessions tended to be taller in high R∶FR. In order to assess these relationships more thoroughly, we examined correlations between the phenotypes (Figure 2A–2C). The high R∶FR and low R∶FR phenotypes were highly positively correlated (*P*-value
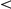
2.2e-16, r = 0.85) and the high R∶FR and response phenotypes were strongly negatively correlated (*P*-value
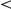
2.2e-16, r = −0.67). The correlation between low R∶FR and response was also significant, although this correlation was much weaker (*P*-value  = 0.016, r = −0.18). These results suggest that much of the variation in the shade avoidance response, as well in hypocotyl height in shade, could be attributed to variation in hypocotyl height in sun conditions, with tall accessions responding less strongly to reduced R∶FR. This relationship, however, was not absolute; analysis of the residuals from a regression of response against height in high R∶FR revealed that the some accessions responded more or less strongly than predicted (Figure 2D). In order to capture this “corrected” variation in response, we used these residuals as a fourth phenotype. Unlike the response phenotype, this corrected response phenotype is significantly correlated with height in low R∶FR (*P*-value = 5.8e-14, r = 0.52) and response to low R∶FR (*P*-value = 2.2e-16, r = 0.75) without being significantly correlated with height in high R∶FR (*P*-value = 1, r = 4.6e-16) (Figure S2). Therefore, the inclusion of this corrected phenotype in our analysis permitted the differentiation of genetic variants that specifically underlie variation in low R∶FR mediated elongation from alleles that underlie general elongation variation.

**Figure 3:**
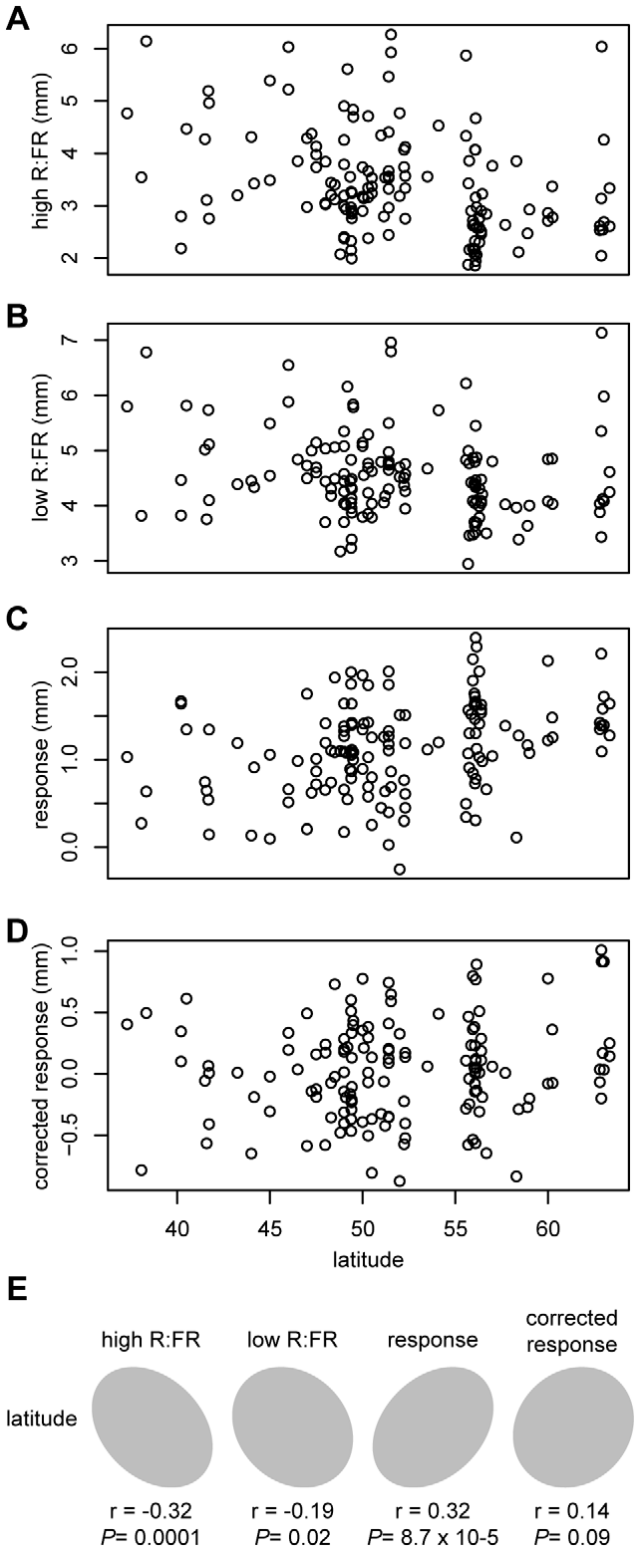
Correlations between phenotypes and latitude. Relationships between phenotypic values and latitude of accession origin for European accessions (A–D) and the correlation ellipses for these comparisons (E).

Previous studies have found negative correlations between hypocotyl height and latitude of accession origin in European *Arabidopsis* accessions [28]–[30], suggesting that this natural variation in light sensitivity could be a result of adaptation to the north-south gradient in ambient light intensity. Since the population structure of *Arabidopsis* in Europe is best thought of as isolation-by-distance [31], confounding due to population structure is a risk when using geographically-correlated phenotypes in GWAS. To test whether phenotypes measured for this study were correlated with latitude, and therefore potentially at risk of population structure problems in GWAS analysis, we examined the relationships between these phenotypes and the latitude of origin for European accessions. Both hypocotyl height in high R∶FR and response to low R∶FR were significantly correlated with latitude (*P*-value = 0.0001 and *P*-value
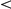
0.0001, respectively)(Figure 3). Hypocotyl height in low R∶FR was also correlated, but with lower significance (*P*-value = 0.021). Although these correlations were not particularly strong (r = −0.32, 0.32, and −0.19, respectively), we still concluded that a population structure correction was necessary in our GWAS study. Interestingly, the corrected response phenotype was not significantly correlated with geography (*P*-value = 0.09), although the ratio of R∶FR light decreases with latitude [32]. This result suggests that variation in the corrected shade avoidance response, if adaptive, might not be due to the same selective pressure responsible for the more generalized differences in light sensitivity seen in *Arabidopsis*. An interesting possibility is that shade avoidance is locally adaptive and is therefore driven more by local variation in plant community composition than by larger-scale patterns of R∶FR. Evidence of local adaptation has been found in *Arabidopsis* [33], and the idea that shade avoidance is locally adaptive would be consistent with the adaptive population-level variation in shade-induced elongation seen in both *Impatiens capensis* [34] and *Abutilon theophrasti* [35].

### Association Mapping

**Figure 4:**
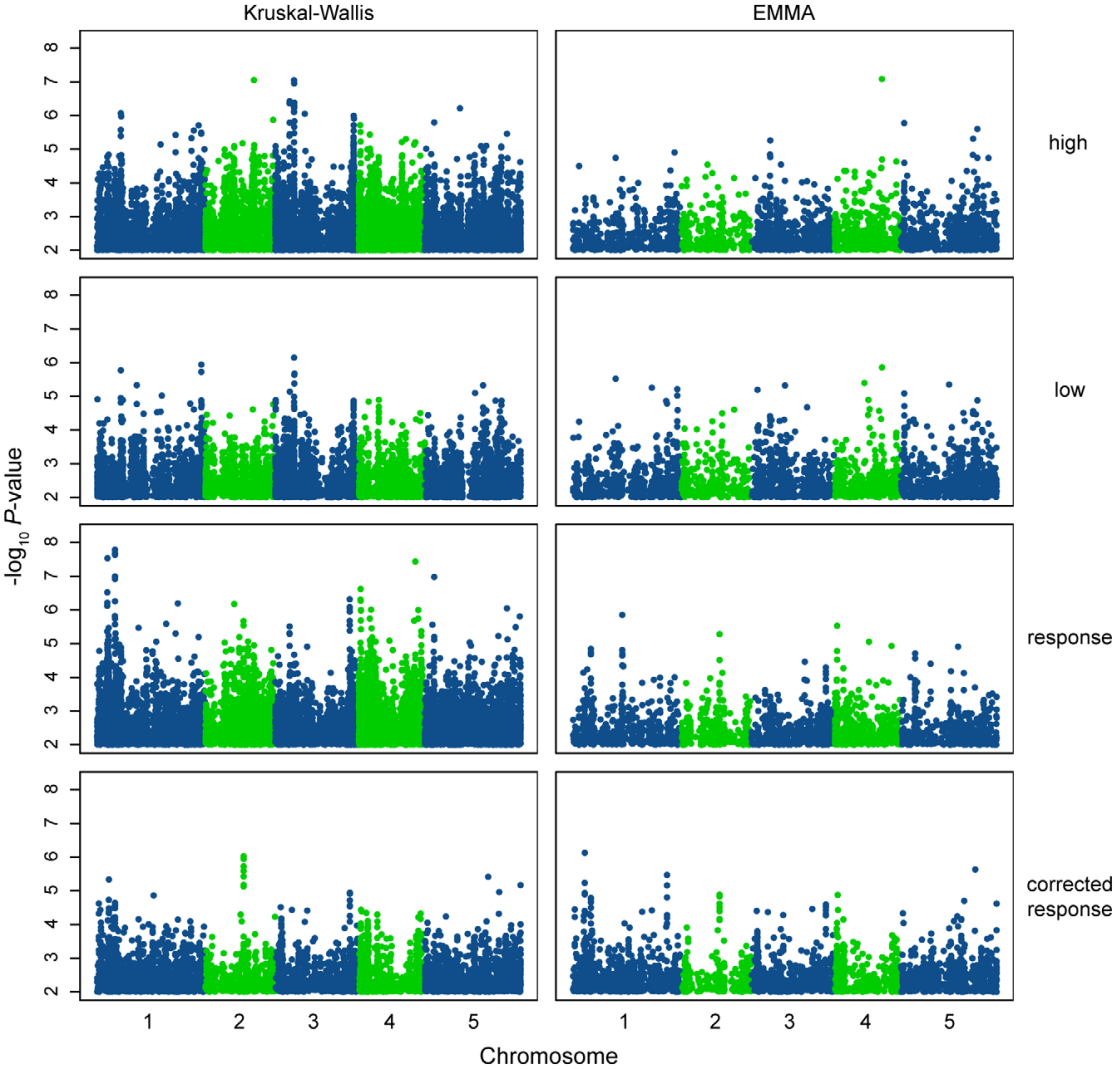
Manhattan plots of GWAS results. Genome-wide distribution of the −log10 *P*-values of SNP/phenotype associations using the Kruskal-Wallis (left panels) and EMMA (right panels) methods. For clarity, only SNPs with a −log10*P*-value 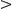 = 2 are shown. Out of 214548 SNPs assayed, 11102, 6864, 11616, and 5301 SNPs are represented in the Kruskal-Wallis panels (top to bottom) while 2538, 2698, 2399, and 2802 SNPs are represented in the EMMA panels (top to bottom). SNPs are accurately plotted according to their position along the appropriate chromosome. Plotting colors alternate between blue and green in order to facilitate the visualization of individual chromosomes.

To uncover the genotypic variation underlying these shade avoidance traits, the significance of associations between phenotypes and the approximately 210,000 genome-wide SNP markers from Atwell et al. [36] was evaluated using both linear mixed model (EMMA) [37] and non-parametric (Kruskal-Wallis) approaches. Genotype information was not available for twelve of the accessions phenotyped for shade avoidance, bringing the total number of accessions used for association testing to 168 (Table S1). When the results of these tests were plotted as genome scans (Figure 4), significant SNPs, some arranged in distinct peaks, were visible for all phenotypes using both association methods. These scans show many peaks of moderate significance rather than the single dominant peak seen in GWAS studies of sodium accumulation and response to bacterial elicitors [36], [38]. This result suggests that variation mapped here is polygenic, as might be expected for an environmentally-sensitive developmental trait (for other examples, see [36]). Comparing across phenotypes, the differences between the significance and location of these peaks allude to differences in the genetic variation underlying the observed variation in the four traits. Differences in peak number and significance were also seen between the two statistical methods, with a greater number of more highly significant peaks seen in the Kruskal-Wallis (KW) scans. The Kruskal-Wallis test includes no correction for population structure, resulting in inflated *P*-values genomewide for all phenotypes, while the use of a kinship matrix to correct for population structure, as implemented in the EMMA method, reduced this *P*-value inflation (Figure S3). Although the Kruskal-Wallis method results in more false positives than the EMMA method, it does have two advantages. First, as a non-parametric test, it is more robust than EMMA. Secondly, since it includes no correction for population structure, the KW method presents no risk of *P*-value overcorrection when applied to traits that are correlated with population structure. Comparisons between the KW and EMMA *P*-values for all SNPs (Figure S4) show that while some SNPs were considered significant in both EMMA and KW tests, the vast majority of SNPs were significant in either one test or the other, most likely for the reasons mentioned above. Therefore, we felt that it was important to consider associations made with both EMMA and KW, keeping the limitations and advantages of both tests in mind.

To assess whether genome-wide associations were a result of “true” signal rather than noise, associations in the genomic region +/−20 kb around the genes *PHYC* and *PHYB* were examined. Natural genetic variation in both of these photoreceptors has previously been shown to underlie variation in hypocotyl elongation in white and red light [21], [30], [39]. Indeed, significant SNPs in linkage disequilibrium with both *PHYC* and *PHYB* were identified using the KW method (maximum −log10 *P*-value in a +/−20 kb window around the genes = 4.83 and 5.09, respectively; Figures S5 and S6) and the predicted effect directions of these SNPs were consistent with those of the polymorphisms identified from previous work (data not shown). However, these SNPs were not identified as significant in EMMA tests (although their *P*-values appeared elevated in comparison to surrounding SNPs). Given the geographical distribution of the high R∶FR phenotype, which broadly mirrors population structure [40], it is possible that these tests were overcorrected in EMMA, resulting in false negatives. Alternatively, it is possible that these associations truly are false positives caused by the confounding effects of population structure. To distinguish between these two possibilities, we genotyped our panel of accessions for the previously-identified *PHYC* and *PHYB* variants and tested associations between these variants and the high R∶FR phenotype. The *PHYC* causative variant was significantly associated with hypocotyl height in high R∶FR using KW yet not with EMMA (Table 2), supporting the hypothesis that EMMA overcorrected a true association. On the other hand, none of the *PHYB* polymorphisms that we tested were significant using either association method (Table 2).

**Table 2:** Associations with previously-identified SNPs in *PHYC* and *PHYB*.

SNP	Kruskal-Wallis	EMMA
*PHYC*	4.43	1.72
*PHYB* Site 1	0.20	0.27
*PHYB* Site 3	1.14	0.09
*PHYB* Site 4	0.70	0.17
*PHYB* Site 7	0.41	0.19
*PHYB* Site 12	1.32	0.08

−log10 *P*-value of Kruskal-Wallis and EMMA associations between hypocotyl height in high R∶FR and candidate SNPs in *PHYC* and *PHYB* identified in Balasubramanian et al. [30] and Filiault et al. [39].

There are a variety of possible explanations for this negative result. First, although statistical tests identified polymorphism three as the most significant of the SNPs tested in Filiault et al. [39], the effect of this specific polymorphism was not functionally verified; it is possible that an alternate SNP is the true causative SNP and would show a significant association if tested. A second possibility is that the genome-wide KW association, while true, is not the same association that was identified in Filiault et al. [39], either due to a polymorphism that does not segregate in the parental accession used in Borevitz et al. [21] or due to differences in light treatment between the experiments. Finally, the GWAS *PHYB* association peak could truly be a false positive. Although the difference between the L*er* and Cvi alleles of *PHYB* was significant in a QTL study and has been verified in transgenics [21], [39], it is possible that this difference does not contribute significantly to hypocotyl height either in a broader population sample or under our study conditions. Although additional work is needed to understand these *PHYB* results, the identification of an experimentally verified natural variant of *PHYC* is evidence that the GWAS is identifying true signal in our data set.

### Identification of *A Priori* Candidate Genes

With the GWAS successfully identifying at least one known natural variant, we next looked for novel genetic variation underlying our phenotypes. The first strategy was to focus on a list of *a priori* genes whose specific roles in vegetative shade avoidance responses have been experimentally confirmed (Table 3). A gene was considered significantly associated with a phenotype if at least one SNP in the genomic region +/−20 kb around the gene had a *P*-value of 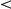
0.0001. The number of SNPs passing this cutoff is provided in Table S2 and detailed descriptions of the individual SNPs used to call *a priori* genes significant can be found in Tables S3 and S4. These criteria were applied to all TAIR9-annotated genes to generate a significant gene list for each combination of phenotype and association method. Fisher exact tests were then used to determine whether the resulting lists were enriched in *a priori* genes. None of the gene lists generated from KW results showed significant enrichment, although associations with *a priori* genes were found for all four phenotypes (Table 3). EMMA results, on the other hand, were significantly enriched in *a priori* candidate genes for both high and low R∶FR phenotypes (*P*-value
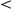
0.016 and *P*-value
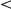
0.005). Given the *P*-value inflation observed when using non-population-structure-corrected KW tests, we expected more false positives with these tests than when using EMMA, potentially explaining the disparity in enrichment *P*-values. Regardless, these results suggested that our GWAS results represented biologically relevant associations rather than noise.

**Table 3:** Associations with *a priori* candidate genes.

Locus	Gene name	KW high	KW low	KW response	KW corrected	EMMA high	EMMA low	EMMA response	EMMA corrected	Pattern category*
AT1G02340	*HFR1*	2.29	2.40	1.77	2.30	1.61	1.24	1.17	1.16	
AT1G04180	*YUCCA9*	2.25	2.06	4.18	4.06	1.56	3.01	2.43	3.03	3
AT1G14920	*GAI*	3.82	2.51	2.98	2.09	1.73	1.76	1.51	1.56	
AT1G65310	*XTH17*	2.6	2.17	2.56	2.74	2.06	3.25	1.86	2.45	
AT1G66350	*RGL1*	2.01	3.29	2.47	3.75	1.12	1.86	1.47	3.03	
AT1G70560	*TAA1*	2.65	3.31	2.01	1.86	3.15	2.92	0.95	1.51	
AT1G70940	*PIN3*	3.32	2.36	3.77	1.66	2.25	1.64	2.34	1.51	
AT1G75450	*CKX5*	2.70	1.99	1.06	1.08	2.59	1.54	1.73	0.66	
AT1G75540	*AtBBX21*	3.15	1.33	2.13	1.65	1.22	1.60	1.90	1.39	
AT2G01570	*RGA*	3.05	2.11	4.12	2.52	1.57	1.20	1.80	1.59	3
AT2G18790	*PHYB*	5.09	2.77	6.18	1.84	2.56	1.56	3.97	1.91	2
AT2G25930	*ELF3*	2.74	2.49	2.15	0.80	1.83	2.22	1.34	1.04	
AT2G32950	*COP1*	2.30	1.87	2.33	3.71	1.76	2.25	1.20	2.30	
AT2G42870	*PAR1*	1.11	1.09	1.27	2.33	1.86	1.80	1.30	1.73	
AT2G43010	*PIF4*	2.49	1.47	3.02	1.46	1.52	1.69	1.39	1.40	
AT2G44910	*ATHB4*	1.91	1.00	3.41	3.25	1.66	1.43	2.27	2.73	
AT2G46970	*PIL1*	2.62	2.48	1.97	2.45	1.9	1.47	2.21	1.95	
AT3G02260	*BIG*	1.87	1.62	1.57	3.19	1.78	2.85	2.01	3.29	
AT3G03450	*RGL2*	4.58	3.23	3.79	1.94	1.46	3.19	1.14	1.68	1
AT3G15540	*IAA19*	7.05	6.15	3.73	0.73	4.84	4.41	2.31	0.87	1
AT3G58850	*PAR2*	3.62	2.21	2.03	1.78	1.43	1.19	1.06	1.49	
AT3G59060	*PIF5*	2.61	3.56	1.05	1.87	2.08	2.64	0.51	1.45	
AT4G13260	*YUCCA2*	1.67	1.82	2.04	2.27	2.14	2.14	2.06	1.66	
AT4G14130	*XTH15*	3.48	3.03	2.61	1.38	2.00	1.51	2.15	1.44	
AT4G16250	*PHYD*	3.02	2.98	3.02	3.36	2.20	2.15	1.43	2.22	
AT4G16780	*ATHB2*	3.39	3.26	3.19	1.84	4.02	4.89	2.27	1.69	1
AT4G18130	*PHYE*	1.74	1.96	2.00	1.97	1.76	1.71	1.47	2.10	
AT4G25420	*GA20ox1*	2.68	2.32	2.68	1.52	4.43	4.33	1.53	1.24	1
AT4G28720	*YUCCA8*	2.61	1.30	2.76	1.12	2.49	2.65	2.32	1.49	
AT4G32280	*IAA29*	5.14	1.73	5.68	1.69	2.19	1.51	2.30	1.24	2
AT4G39400	*BRI1*	1.96	2.23	1.80	1.45	2.21	1.57	2.01	0.81	
AT5G17490	*RGL3*	1.42	1.56	1.21	1.12	1.35	1.91	0.91	1.28	
AT5G43890	*YUCCA5*	3.38	2.40	3.79	2.89	2.61	1.70	4.13	2.59	3
AT5G51810	*GA20ox2*	4.22	4.55	3.21	2.23	3.80	4.47	1.47	2.29	1
AT5G61380	*TOC1*	2.68	2.08	3.91	1.88	1.66	1.84	2.09	1.68	

−log10 *P*-value of most significant Kruskal-Wallis and EMMA associations between hypocotyl phenotypes and *a priori* candidate genes.

*: Significance pattern categories: 1 = general control of hypocotyl height, 2 = control of shade avoidance via hypocotyl height, 3 = specific control of shade avoidance response.

Next, the individual *a priori* candidate gene associations were examined in more detail, with the goal of looking for patterns in the genetic control of phenotypic variation. When the significance of both KW and EMMA associations across the four phenotypes was considered, candidate genes seemed to fall into three main patterns (Table 3 and Figure S7, row1), which corresponded to the phenotypic correlation patterns observed in Figure 2 and Figure S2. The first pattern consisted of genes associated with hypocotyl height under high and/or low R∶FR conditions without showing significant associations with response or corrected response phenotypes. These genes could be responsible for variation in general elongation without causing variation in shade avoidance. Even though *a priori* genes were chosen specifically as known shade avoidance loci, five of the ten significantly-associated genes fell into this generalist category. The functions of these five genes are quite diverse; *GA20ox1* and *GA20ox2* are involved in gibberellic acid (GA) biosynthesis [19], *IAA19* is part of the auxin signaling pathway [41], *RGL2* encodes a DELLA protein involved in the integration of the GA and light signaling pathways [42], and *ATHB2* is a transcription factor involved in phytochrome B signaling [43]. Notably, while all five genes have been shown to be upregulated in response to low R∶FR, their expression under high R∶FR conditions has also been demonstrated [11], [18], [19], suggesting a mechanism whereby variation in these genes could potentially underlie variation in elongation in a more general fashion.

The other two patterns of significance observed involved either the response or the corrected response to low R∶FR. The first of these patterns was association with both height in high R∶FR and response to low R∶FR without a significant association with the corrected R∶FR response. Candidate genes which fit this pattern might underlie variation in the shade avoidance response primarily by controlling hypocotyl height in sun conditions, reflecting the high inverse correlation between these two phenotypes (Figure 2). Two *a priori* candidate genes fell into this category: the auxin-responsive transcription factor *IAA29*, which has been shown to be responsive to both red and far-red light [44], and the photoreceptor PHYB, the primary photoreceptor involved in sensing the changes in R∶FR that initiate shade avoidance [2]. Genes specifically affecting the shade avoidance response would be predicted to fall into the final pattern of significance: significant association with response to low R∶FR and/or corrected response to low R∶FR without a significant association with high R∶FR. Three *a priori* genes *YUCCA5*, *YUCCA9*, and *RGA1* exhibited significance patterns consistent with this third group of genetic control. *YUCCA5* and *YUCCA9* are involved in auxin biosynthesis [15], while *RGA1* is another member of the five-gene *DELLA* family discussed above [20].

One candidate gene that was not significantly associated with our phenotypes was *ELF3*. Although natural variation between the Bayreuth and Shahdara alleles of *ELF3* has been shown to underlie variation in shade avoidance between these two accessions [22], [23], we found no evidence of associations with this variant in our data. This result is perhaps not unexpected as the polymorphism presumed to cause reduced response to shade in the Shahdara accession seems to be rare [23], a condition which would result in very little power to detect this polymorphism in GWAS. Overall, however, the strategy of using an *a priori* gene list was useful one for two reasons. First, significant enrichment of *a priori* genes lends additional support to the hypothesis that the GWAS is indeed identifying true positives. Second, the resulting lists of significantly-associated *a priori* genes and their corresponding significance pattern groups can easily be used to generate specific testable hypotheses about both the identity and molecular nature of natural variants.

### Identification of *De Novo* Candidate Genes

Our final goal was to look beyond our *a priori* gene list to find *de novo* candidates. As in the *a priori* analysis, genes +/−20 kb of a significant SNP were considered significant, but for *de novo* discovery, a more stringent *P*-value cutoff was instituted for KW tests (*P*-value
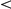
0.00001). This cutoff *P*-value was chosen to be slightly lower than that of the association between height in high R∶FR and *PHYC*, since this association was considered confirmed. For EMMA tests, a cutoff that resulted in a similar number of significant genes for both EMMA and KW tests was chosen (*P*-value
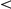
0.0001). SNPs with a minor allele frequency 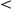
0.1 were also removed from the analysis, since these SNPs can produce misleading results in EMMA tests; this filter reduced the number of SNPs considered from 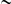
210 k to 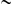
170 k. The number of SNPs matching these criteria is provided in Table S2 and detailed descriptions of the individual SNPs used to call *de novo* genes significant can be found in Datasets S1 and S2. Applying these selection criteria, we identified significant SNPs for all phenotypes (Table S2), defining 1709 genes as significant. As in the *a priori* gene analysis, genes identified using the *de novo* criteria were easily separable into the same three significance pattern groups (Figure S7, row 2). Of the unique genes identified, 192 were significant for both KW and EMMA. Although genes that were significant in both KW and EMMA were considered particularly interesting, all associated genes were included when looking for *de novo* candidates.

To sort through this gene list, we took advantage of metadata to help identify possible *de novo* candidate genes. First, microarray data from previously-published experiments [10], [14] was reanalyzed to generate a list of genes differentially regulated in response to low R∶FR treatment. Secondly, the biological process GO terms and other annotation for all the genes on the list were retrieved. No significant enrichment either for differential regulation or for specific GO terms was seen in this list, nor was any GO term significantly different either between EMMA and KW *de novo* candidate gene lists or among the lists of candidate genes for the four different phenotypes (data not shown). This lack of GO term enrichment is not surprising given both the incomplete nature of the GO resource and the presumed low ratio of causative to non-causative genes in the analysis, resulting both from the +/−20 kb window used for candidate gene identification and from lack of population structure correction in KW tests. Both differential regulation and GO terms were, however, used to manually parse through this comprehensive *de novo* gene list to identify potentially interesting candidates. This selection process reduced the *de novo* candidate list to 53 genes which were subsequently easily assigned to the three significance pattern groups established in the *a priori* analysis (Figure S7, third row). The resulting *de novo* gene list is in Table S5.

Although this list of candidate genes included many *a priori* genes, we were able to identify truly *de novo* candidates, as well. From the filtered list of 53 *de novo* genes, 28 genes fell into the first significance group pattern: genes responsible for general hypocotyl height variation. Here, two genomic regions stood out as being significant in both KW and EMMA tests. The first region contained *IAA19*, which had been found as an *a priori* candidate gene, and the second region contained *CGA1*. This low R∶FR-regulated GATA family transcription factor functions downstream of the *DELLA*s to control elongation growth, and its expression is increased in *pif*-family (*PHYTOCHROME INTERACTING FACTOR*) knockout plants. This regulation seems to be direct, since an element of the *CGA1* promoter co-immunoprecipitates with PIF3 [45]. Given this *de novo* association, as well as those of two *DELLA*s (*RGA* and *RGL2*) seen in the *a priori* analysis, it seems that modulating the integration of light and GA signals could be a common mechanism for generating general hypocotyl height variation in natural populations.

Genetic variants of the loci in the second significance pattern group are hypothesized to cause hypocotyl height-dependent variation in shade avoidance. Two of the seven *de novo* genes in this group, *PHYB* and *IAA29*, had also been analyzed as *a priori* genes. The only group two gene to be significant in both EMMA and KW tests, however, was a locus near, yet not in the same +/−20 kb window as, *IAA29*. This gene is *ATH1* (AT4G32980), a homeobox transcription factor implicated in photomorphogenesis [46] that has also been shown to be involved in stem growth and shoot apical meristem maintenance in older plants, as well [47], [48].

Significance group three contains genes with variants that potentially influenceshade avoidance in a specific manner. Interestingly, many group three *de novo* genes seemed to be involved in phytochrome A signaling. *PHYA* and *PIF3*, a transcription factor that interacts directly with both phyA and phyB, are separated only by about 16 kb in the *Arabidopsis* genome. A significantly-associated SNP fell into this interval, making both of these genes potential *de novo* candidates. Two additional genes involved in phyA signaling, *ATNAP2/ABCI21* and *FRS11* [44], [49] were also significant. A fifth gene, *PP5Pa*, a proposed inorganic pyrophosphatase, initially seemed an unlikely candidate despite being differentially-regulated in response to low R∶FR and being significant in both EMMA and KW tests. However, transcription of this gene has been shown to be under the control of *FAR1* [50], a transcription factor in the phytochrome A signaling pathway that is involved in the nuclear accumulation of phyA [51]. Altogether, five of 18 group three *de novo* candidate genes are involved in phyA signaling, suggesting that variation in this pathway could be responsible for at least some of the observed variation in the shade avoidance response. This phenomenon could be explained by the light-labile nature of phyA itself. Although phytochrome A is rapidly degraded in red light, it becomes more stable as the ratio of R∶FR decreases, allowing increased signaling through the phyA pathway and a concomitant inhibition of elongation growth in shade conditions [52].

Variants in both *PHYA* and *PHYB* were associated with variation in shade avoidance, yet the association/phenotype significance patterns seemed to suggest that *PHYB* variation affected shade avoidance in a strictly height-dependent way, while *PHYA* control was more specific for the response itself. We decided, therefore, to ask whether these two variants exerted independent effects on our phenotypes. Two-way ANOVAs with the most significant *PHYB* and *PHYA* SNPs as factors found no significant interaction term for any of the four phenotypes used in this study (data not shown), and the loci seemed to be acting additively (Figure S8). These results indicate that genetic variants linked to *PHYA* and *PHYB* were exerting independent control over shade avoidance. *T*-tests between the means of the allelic variants of both SNPs showed effect sizes and *P*-values that were consistent with the patterns seen in the GWAS (Table S6). The notion that *PHYA* and *PHYB* act independently in shade avoidance is in agreement with a microarray study of shade avoidance using *phyB* and *phyAB* mutants which identified a number of shade-responsive genes under independent control of *PHYA* [53]. Again, however, *PIF3* and *PHYA* are situated quite nearby each other in the genome, so the SNP identified as significant here could be a marker for variation in either gene. Nonetheless, this particular association is one of many promising targets for which validation of these GWAS results using crosses and functional studies seems warranted.

Finally, intrigued by the result that variation in *PHYA/PIF3* seemed to underlie specific shade avoidance variation, we asked whether the *PHYA/PIF3* variant could shed light on the idea that the shade avoidance response could be locally adaptive. We decided to focus on Swedish accessions, since 43 of the 168 accessions used for the GWAS study originated from Sweden. When the phenotypes of these accessions were plotted on a map (Figure S9A–S9D), no obvious geographic patterns could be seen and in fact, none of the phenotypes were significantly correlated with latitude (data not shown). On the contrary, phenotypes from accessions in very close proximity to one another often had quite disparate phenotypes, especially for the response and corrected response phenotypes. This observation is consistent with the population-level phenotypic variation that could result from adaptation to local R∶FR conditions. The allelic distribution of the most significant *PHYA/PIF3* SNP showed a similar pattern of local co-existence, with the exception of the 14 most northern accessions which all carried the same variant (Figure S9E). *T*-tests for differences between the mean phenotypes of the two alleles were performed for all four phenotypes (Figure S9F). These tests indicated that just as in the main set of accessions, the *PHYA/PIF3* variant had a specific effect on the shade avoidance response in the Swedish subset. Given that *Arabidopsis* exhibits isolation by distance [31], we cannot rule out the possibility that these associations are false positives due to population structure, especially since the most northern accessions all carry the same allele of the SNP under consideration. However, if the variation in shade avoidance that has been measured in this study is indeed adaptive, then the evidence presented here is a solid starting point for further exploration of hypothetical local adaptation in shade avoidance in Swedish *Arabidopsis* populations.

### Conclusions

We performed a genome-wide association study (GWAS) to look for genetic variants underlying four phenotypes: hypocotyl height in both high and low R∶FR, the response of hypocotyl height to shade, and the response to shade corrected for hypocotyl height. Rather than the few peaks of large effect size seen in some earlier published *Arabidopsis* GWAS, our results showed many peaks with small to moderate effect sizes. Instead of representing a shortcoming with the study or method, these results suggest that variation in the shade avoidance response is complex trait that is controlled by many genetic variants. Through analysis of known variants, *a priori* candidate genes lists, and metadata-enabled *de novo* candidate discovery, we were able to identify genetic variants associated with shade avoidance phenotypes. One goal of future work will be to verify these associations in lines that minimize confounding due to population structure, such as F2 populations or recombinant inbred lines. A second goal will be to identify and characterize causative polymorphisms through functional molecular work.

While previous GWAS studies in *Arabidopsis* have found environment-dependent associations [54]–[56], the results of the study described here emphasize the strength of explicitly incorporating GxE interactions into the GWAS approach. First, our study design enabled the identification of genetic variants specifically underlying the response to low R∶FR. As many aspects of plant development and physiology are intrinsically environmentally-sensitive, an improved understanding of genotype-by-environment interactions will be a key part of exploring the genotype-phenotype map for these traits. As statistical methods and mapping designs improve [57], our power to examine these interactions will only continue to grow.

The second benefit of our study design is that the results serve as a springboard to ecological and population genetics studies exploring the evolutionary relevance of environmental responses. For example, in this study, the observation that the shade avoidance response is not correlated with latitude lead us to hypothesize that the response to low R∶FR is locally adaptive. Our subsequent GWAS identified variants that were specifically associated with the shade avoidance response, suggesting a set of experiments that can be performed to explore this hypothesis. First, our candidate variant list can be used in designing physiological and/or genetic manipulations to assess whether this variation in shade avoidance is an example of adaptive plasticity [58], [59]. Second, the resequencing of hundreds of *Arabidopsis* accessions [60] will provide a powerful resource to look for genomic evidence of selection around candidate SNPs. Finally, if the variants identified in our GWAS are adaptive, it would be interesting to understand the scale of this adaptation. Since little information about habitat or ecology was collected at the time of accession sampling, this work would require returning to the field, assessing the light environment in field sites and taking new population samples. If our candidate SNPs are responsible for local adaptation, then population-level differences in the frequency of these variants should correspond with local differences in the R∶FR ratio. This suite of experiments, which has the potential to shed light on the genetics of phenotypic plasticity, is made possible by the specific nature of the candidate SNP lists generated as a result of the incorporation of genotype by environment interactions into GWAS, indicating that this strategy promises to be a useful tool in furthering our understanding of evolution and natural variation.

## Materials and Methods

### Plant Culture and Measurement

180 *Arabidopsis thaliana* accessions (Table S1) were phenotyped. Seeds were gas sterilized, plated on 0.5× MSMO with 0.7% agar, and stratified for four days in the dark at 4

C. Plates were then moved to two LED chambers with constant light conditions set to 34 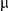
E/m^2^/s red light and 7 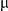
E/m^2^/s blue light. After 24 hours, far-red light was added to bring the red-to-far-red ratio (R∶FR) to 2. After an additional 24 hours, non-germinated seeds were marked and excluded from further analysis in order to minimize hypocotyl height variation due solely to variation in germination time. 24 hours after this marking, the R∶FR ratio in one chamber was lowered to 0.5 and plants were grown for an additional 4 days. Seedings were harvested to transparencies and scanned to .jpg files. Hypocotyl height was measured from these images using Image J [61]. A completely randomized design of two repetitions of 20 plants each per treatment was used. The experiment was repeated with a reversal of the R∶FR treatment assignments for the two chambers.

### Phenotype Modeling

This, and all subsequent analyses were done using the R statistical programming language [62]. Phenotypes were modeled with the following mixed linear model using the lme4 package [63]:
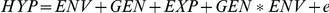
where *HYP* is hypocotyl height, *ENV* is light treatment (high or low R∶FR), *GEN* is genotype (accession), *EXP* is experimental repeat, GEN*ENV is the genotype by environment interaction, and *e* is the error. *ENV* is fitted as a fixed effect while all other variables are fitted as random effects. Significance of each model term was assessed using the anova.lm method implemented in R. The predicted effects for hypocotyl height in high and low R∶FR, as well as for response to R∶FR were extracted and used as phenotypes in GWAS analysis.

To determine heritability of hypocotyl height, the following model was fit for both high and low R∶FR data:
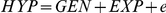
where *HYP* is hypocotyl height, *GEN* is genotype (accession), *EXP* is experimental repeat, and *e* is the error, with all variables fitted as random effects. Heritability was then calculated as the genotypic variance divided by the total variance.

Genetic correlation across high and low R∶FR environments was calculated as in Falconer and MacKay [64] using variance estimates from the above models.

### Genome-Wide Associations

All analyses were done in R [62]. Two methods were used to perform association tests between modeled phenotypes and the genome-wide SNP data from Atwell et al. [36]. The first method was EMMA, the mixed linear model approach with a K matrix as populations structure correction as outlined in Kang et al. [37], and the second method was a Kruskal-Wallis test. Linkage disequilibrium was calculated using the genetics package [65]. Phytochrome B and C genotyping was done as in Balasubramanian et al. [30] and Filiault et al. [39], with results in Table S7.

### Enrichment Analysis

Genes differentially regulated in response to R∶FR treatment were determined by reanalyzing data from Sessa et al. [10] and Tao et al. [14] using the limma package [66] in Bioconductor [67]. A false discovery rate (FDR) cutoff of 0.1 was used for determining gene significance. GO annotation and other annotation was taken from the org.At.tairGO package [68] in Bioconductor [67]. Enrichment for *a priori* genes and for R∶FR differentially-regulated genes was assessed with a Fisher's exact test. The GOstat program [69] with default settings, an FDR of 0.05, and the TAIR GO database was used to look for overrepresentation of GO terms in candidate gene lists.

## Supporting Information

Dataset S1A .csv file providing the minor allele frequency(MAF), −log10 *P*-value, and *P*-value rank of all SNPs with a KW *P*-value
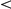
0.00001.(CSV)

Dataset S2A .csv file providing the minor allele frequency(MAF), −log10 *P*-value, *P*-value rank, effect size, and EMMA variance components of all SNPs with an EMMA *P*-value
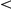
0.0001.(CSV)

Figure S1 Distributions of phenotypes derived from the mixed effects model. Histograms of the fitted values for hypocotyl height in high R∶FR (A), height in low R∶FR (B), and response to low R∶FR (C). The distribution of the corrected response phenotype is shown in Figure 2D.
(PDF)

Figure S2 Correlations with the corrected response phenotype. Correlations between the corrected response phenotype and hypocotyl height in high R∶FR (A), height in low R∶FR (B), and response to low R∶FR (C).
(PDF)

Figure S3 Q-Q plots. Quantile-quantile plots of Kruskal-Wallis and EMMA *P*-values for all four phenotypes showing the distribution of observed *P*-values (black dots) compared to the expected *P*-value distribution (red lines). The upward shift of observed *P*-values away from the diagonal represents *P*-value inflation.
(PDF)

Figure S4 Comparison of *P*-values between Kruskal Wallis and EMMA tests. Scatter plots comparing −log10 *P*-values for Kruskal-Wallis (KW) and EMMA tests for all four phenotypes. Shaded boxes delimit SNPs that are considered significant for *de novo* candidate gene discovery. The green boxes contain SNPs significant in EMMA only, SNPs in the purple boxes are significant for KW only, and the pink boxes denote SNPs that are significant for both tests. The numbers printed within each box represent the number of SNPs in each box. The number of points in each box may not match this number exactly due to overplotting of SNPs with identical or nearly-identical *P*-values.
(PDF)

Figure S5Detailed view of associations with high R∶FR around *PHYC*. The lower panel is a detailed view of the area highlighted by the green box in the upper panel. In both panels, open circles indicate the −log10 *P*-value of the SNPs in the region. Blue circles represent EMMA *P*-values while red circles represent Kruskal-Wallis *P*-values. Green rectangles running horizontally through the lower panel represent the genes +/−20 kb around *PHYC*. The pairwise linkage disequilibrium (R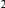) between SNPs is indicated below the genes in the lower panel, with darker colors representing higher linkage disequilibrium.(PDF)

Figure S6Detailed view of associations with high R∶FR around *PHYB*. The lower panel is a detailed view of the area highlighted by the green box in the upper panel. In both panels, open circles indicate the −log10 *P*-value of the SNPs in the region. Blue circles represent EMMA *P*-values while red circles represent Kruskal-Wallis *P*-values. Green rectangles running horizontally through the lower panel represent the genes +/−20 kb around *PHYB*. The pairwise linkage disequilibrium (R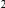) between SNPs is indicated below the genes in the lower panel, with darker colors representing higher linkage disequilibrium.(PDF)

Figure S7 Venn diagrams of candidate gene lists. Venn diagrams showing the number of significant genes common to all combinations of the four study phenotypes. Diagrams for both Kruskal-Wallis and EMMA tests for the three candidate gene lists described in the text are presented.
(PDF)

Figure S8 Phenotypes of accessions carrying the most significant SNPs around *PHYA* and *PHYB*. Box plots for all phenotypes. The four groups in each plot represent the four possible allelic combinations of the most significantly-associated SNPs around *PHYA* and *PHYB*. The *PHYA* SNP is Chr1:3079229 and the *PHYB* SNP is Chr2:8139482 (TAIR 9 annotation). The letter A in each genotype group designation denotes the *PHYA* genotype, while the letter B denotes the *PHYB* genotype.
(PDF)

Figure S9 Phenotypes and *PHYA/PIF3* variation in Swedish accessions. (A–D) Geographic distribution of phenotypic values. Phenotypic values are represented by a gradient in both size and color; small blue circles represent smaller values, while large red circles indicate larger values. (E) Geographic distribution of the alleles of the most significant SNP near *PHYA/PIF3* (Chr1:3079229) for the Swedish accessions used in this study. (F) Box plots for all phenotypes grouped by the alleles represented in panel E. *T*-test *P*-values for differences in trait means between the alleles are presented above each box plot.
(PDF)

Table S1 *Arabidopsis thaliana* accessions used in this study.
(PDF)

Table S2 Number of SNPs consided significant for all cutoff criteria used in this study.
(PDF)

Table S3 Characterization of the significant SNPs identified in *a priori* Kruskal Wallis tests, including position, minor allele frequency, and *P*-value rank.
(PDF)

Table S4 Characterization of the significant SNPs identified in *a priori* EMMA tests, including position, minor allele frequency, *P*-value rank, effect size, and EMMA variance components.
(PDF)

Table S5 GWAS results for loci selected as interesting *de novo* candidate genes.
(PDF)

Table S6 Difference in allelic means and *t*-test *P*-values for the most significant *PHYA* and *PHYB* SNPs.
(PDF)

Table S7 Phytochrome B and C genotyping results.
(PDF)
