# Supplementary figures and images for: A Genome-Wide Association Study Identifies Variants Underlying the Arabidopsis thaliana Shade Avoidance Response
Source: PLoS Genet. 2012 Mar 15;8(3):e1002589. doi: 10.1371/journal.pgen.1002589 (PMC3305432; doi:10.1371/journal.pgen.1002589)

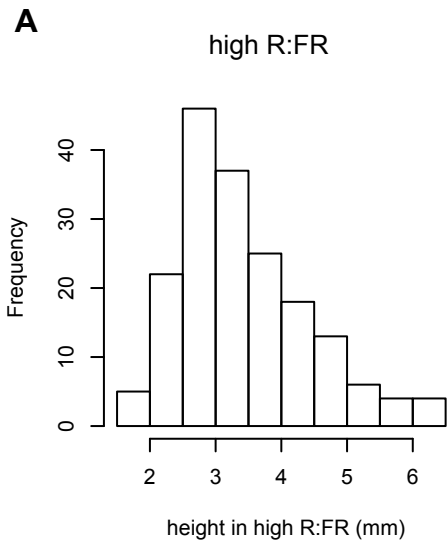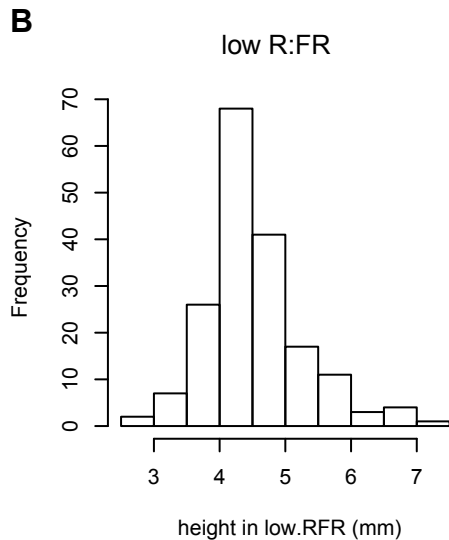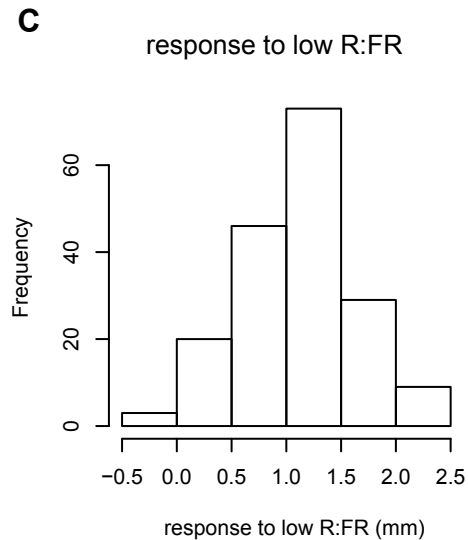

Supplement: Figure S1 — Distributions of phenotypes derived from the mixed effects model. Histograms of the fitted values for hypocotyl height in high R∶FR (A), height in low R∶FR (B), and response to low R∶FR (C). The distribution of the corrected response phenotype is shown in Figure 2D. (PDF) [file pgen.1002589.s003.pdf]

KW

high R:FR

low R:FR

response

corrected response

Observed  $-\log_{10} P$ -value

EMMA

Expected  $-\log_{10} P$ -value

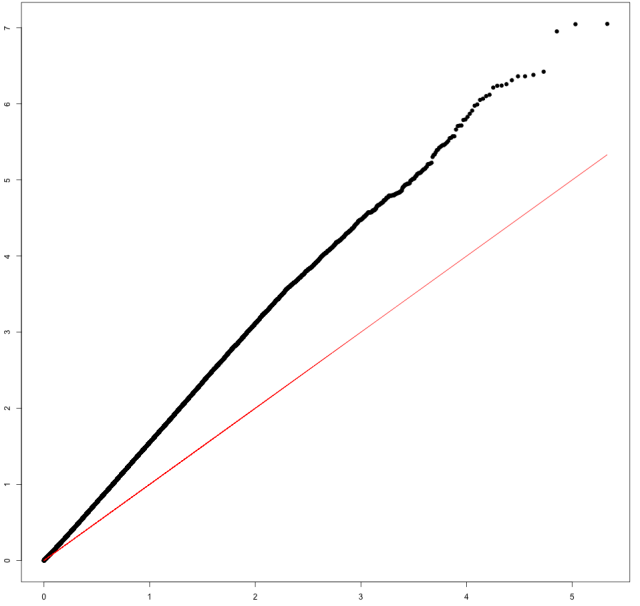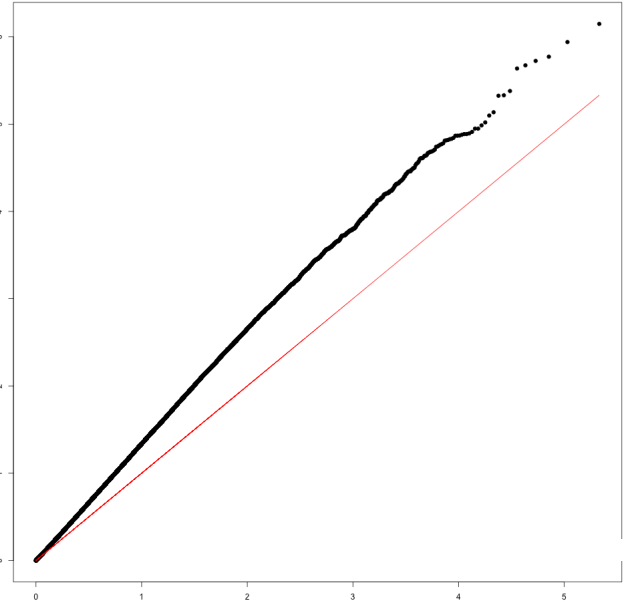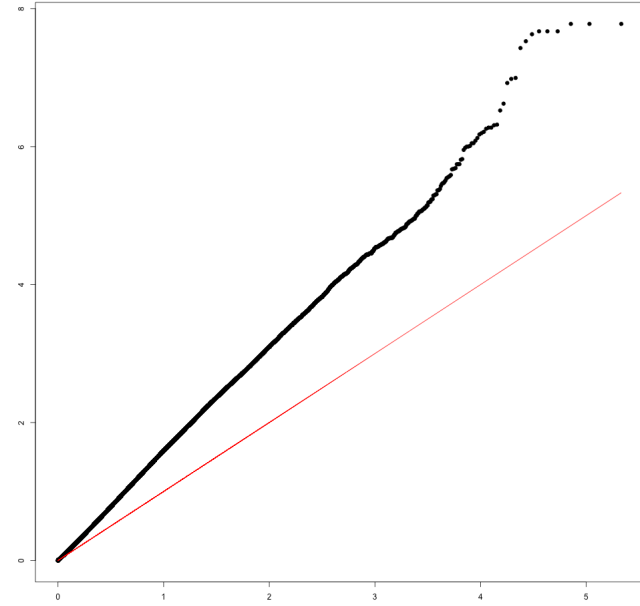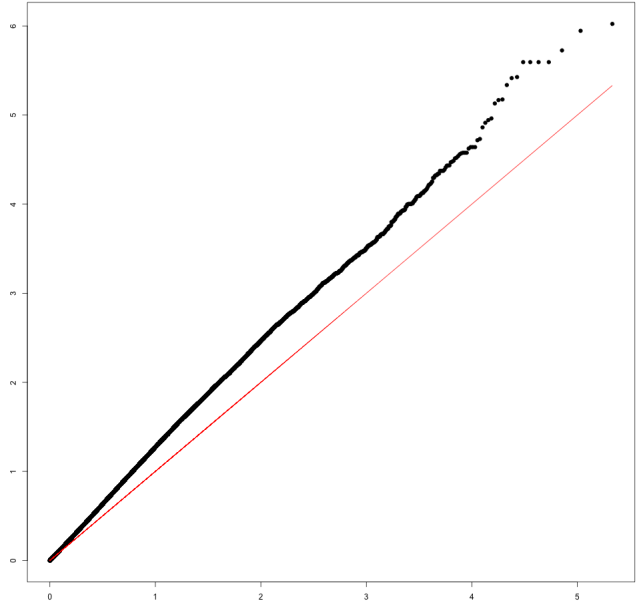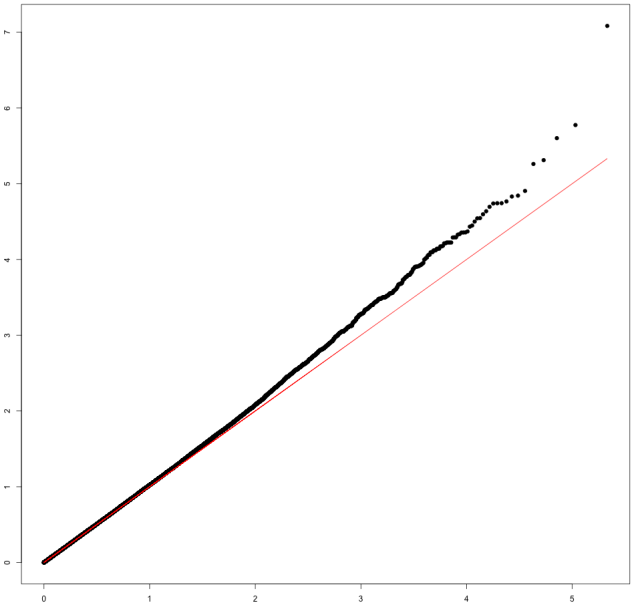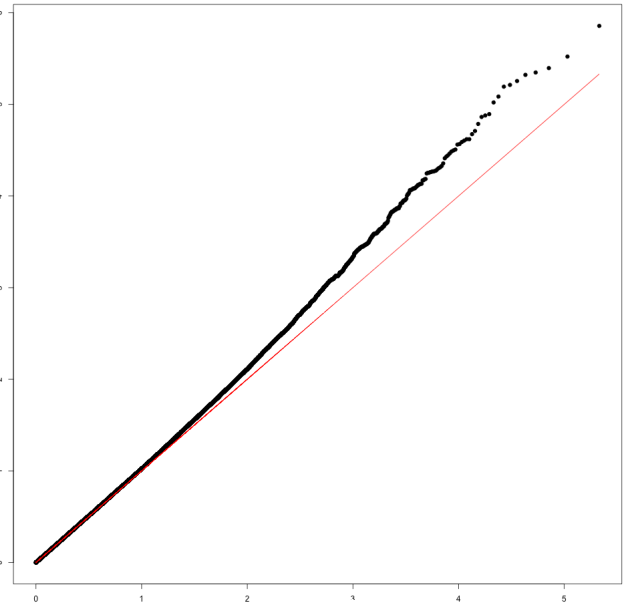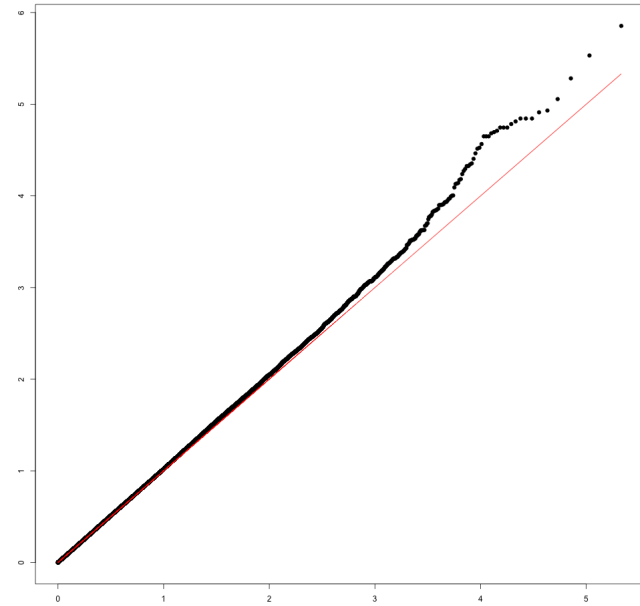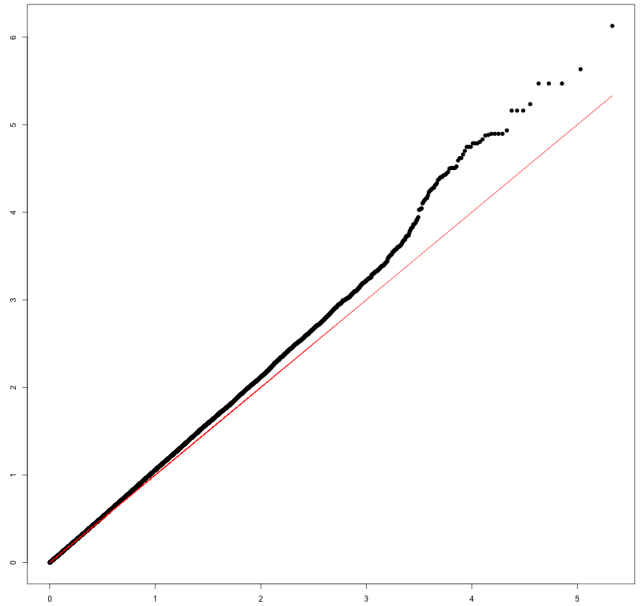

Supplement: Figure S3 — Q-Q plots. Quantile-quantile plots of Kruskal-Wallis and EMMA P-values for all four phenotypes showing the distribution of observed P-values (black dots) compared to the expected P-value distribution (red lines). The upward shift of observed P-values away from the diagonal represents P-value inflation. (PDF) [file pgen.1002589.s005.pdf]

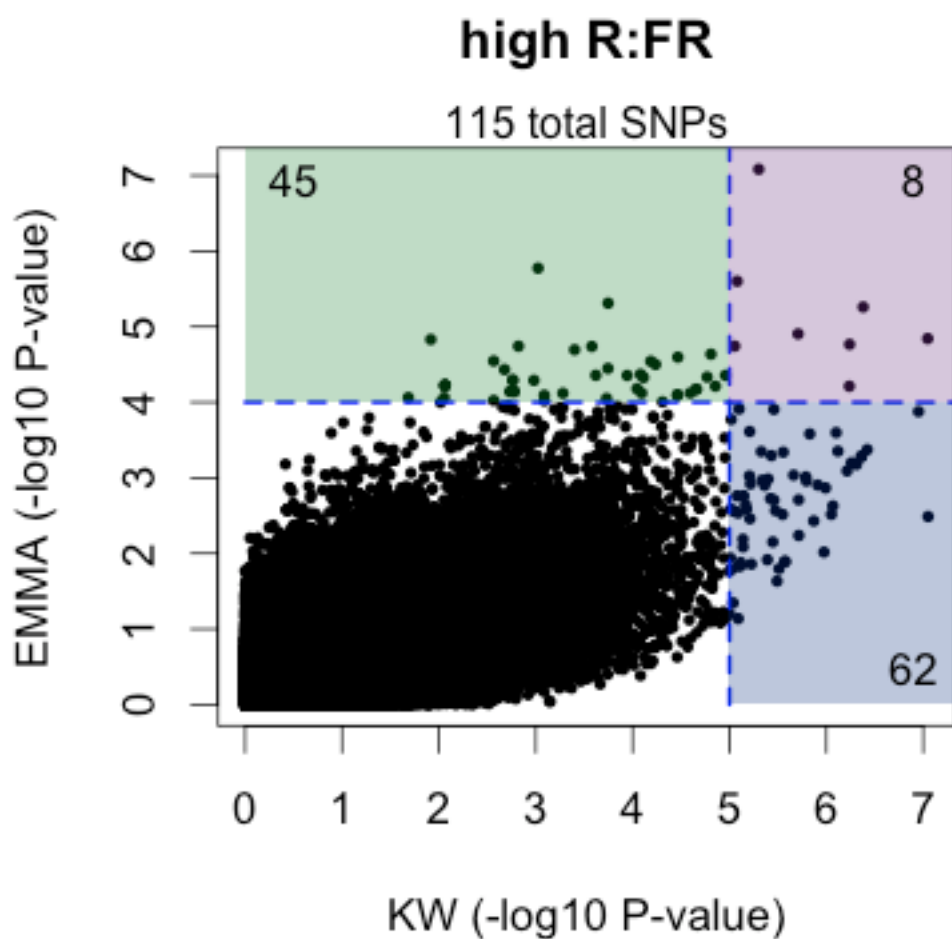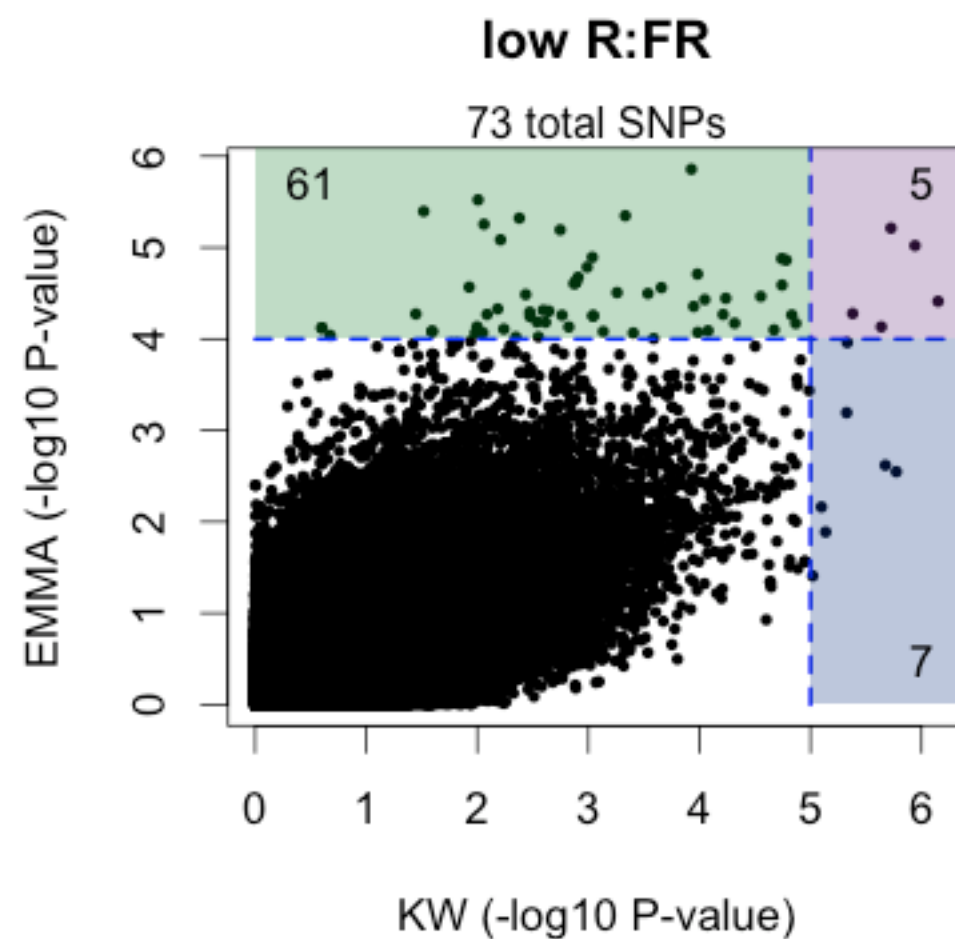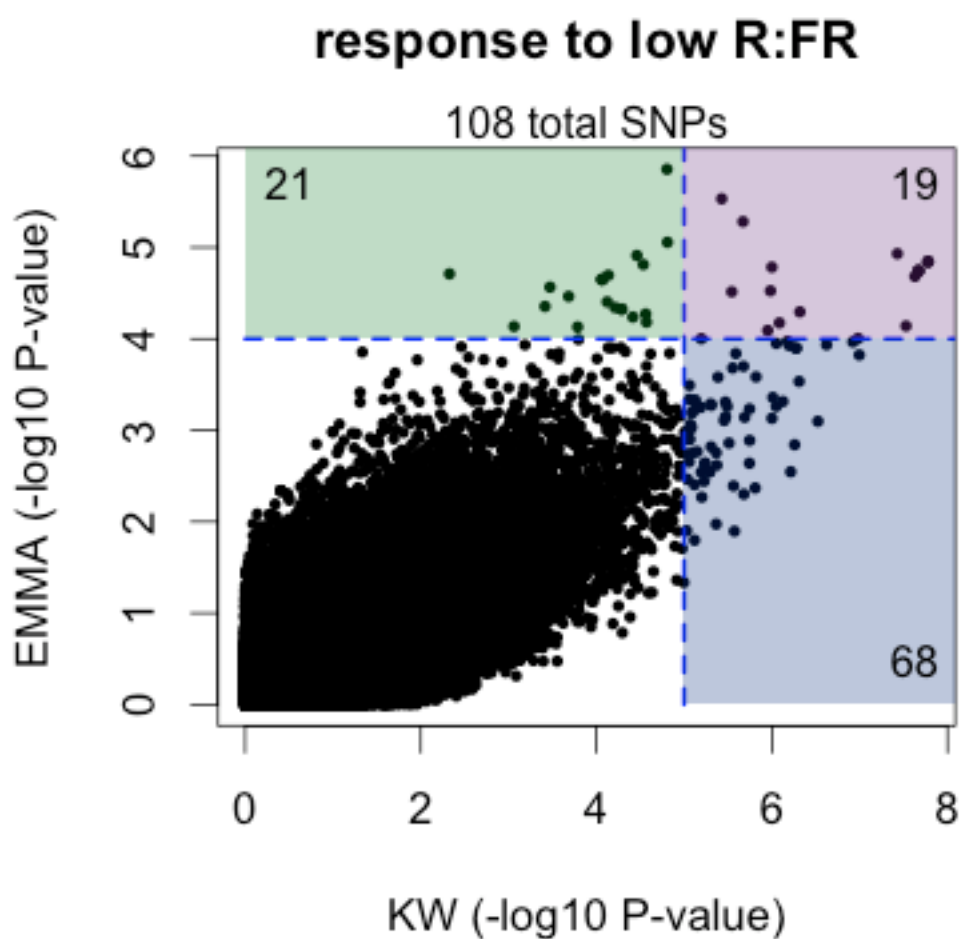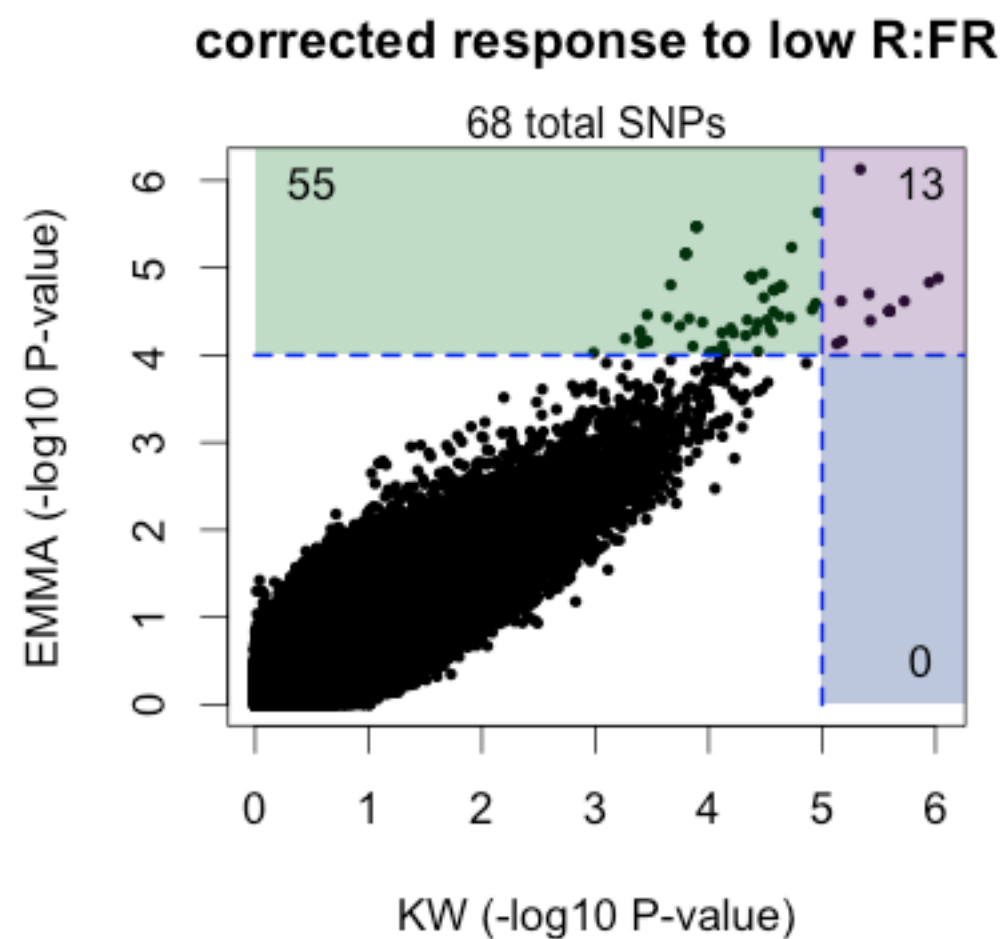

Supplement: Figure S4 — Comparison of P-values between Kruskal Wallis and EMMA tests. Scatter plots comparing −log10 P-values for Kruskal-Wallis (KW) and EMMA tests for all four phenotypes. Shaded boxes delimit SNPs that are considered significant for de novo candidate gene discovery. The green boxes contain SNPs significant in EMMA only, SNPs in the purple boxes are significant for KW only, and the pink boxes denote SNPs that are significant for both tests. The numbers printed within each box represent the number of SNPs in each box. The number of points in each box may not match this number exactly due to overplotting of SNPs with identical or nearly-identical P-values. (PDF) [file pgen.1002589.s006.pdf]

# PHYC - AT5G35840

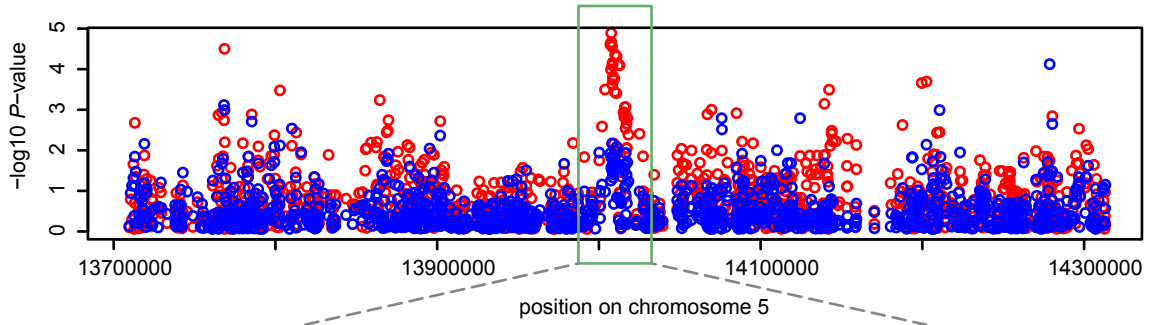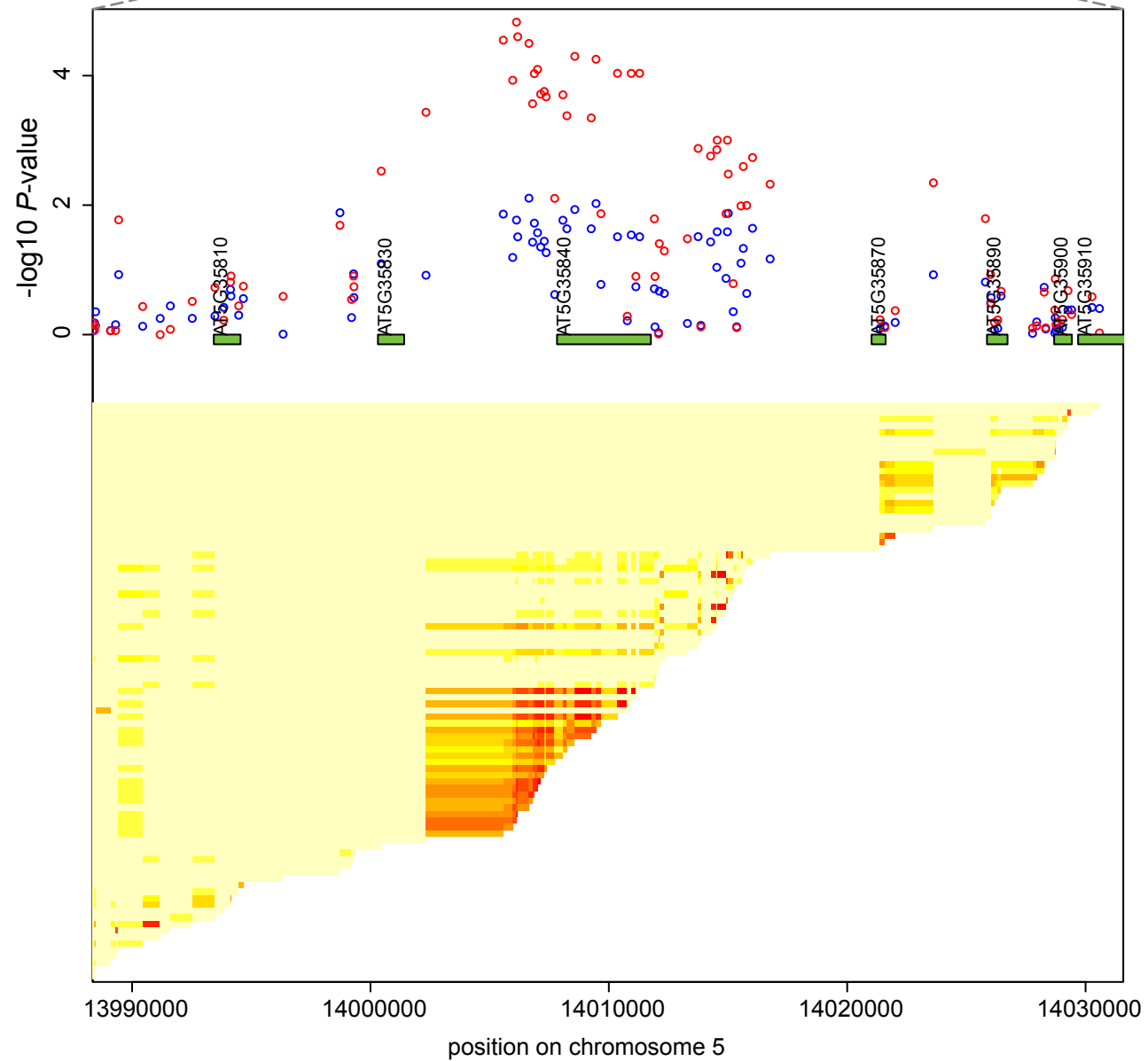

Supplement: Figure S5 — Detailed view of associations with high R∶FR around PHYC. The lower panel is a detailed view of the area highlighted by the green box in the upper panel. In both panels, open circles indicate the −log10 P-value of the SNPs in the region. Blue circles represent EMMA P-values while red circles represent Kruskal-Wallis P-values. Green rectangles running horizontally through the lower panel represent the genes +/−20 kb around PHYC. The pairwise linkage disequilibrium (R) between SNPs is indicated below the genes in the lower panel, with darker colors representing higher linkage disequilibrium. (PDF) [file pgen.1002589.s007.pdf]

# PHYB - AT2G18790

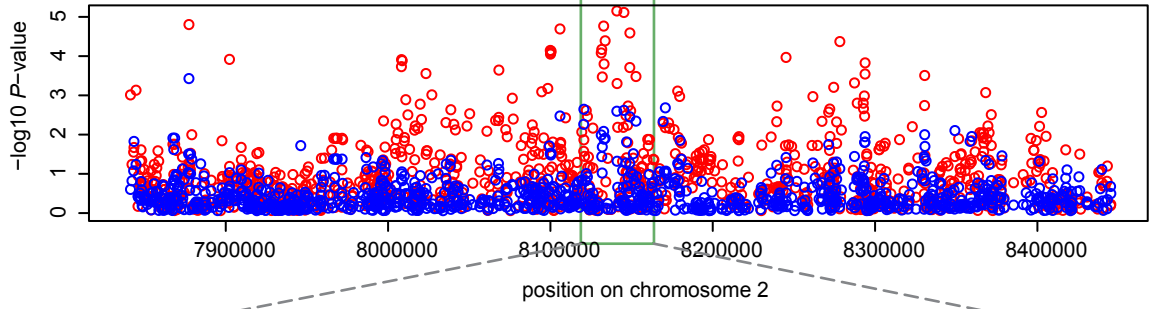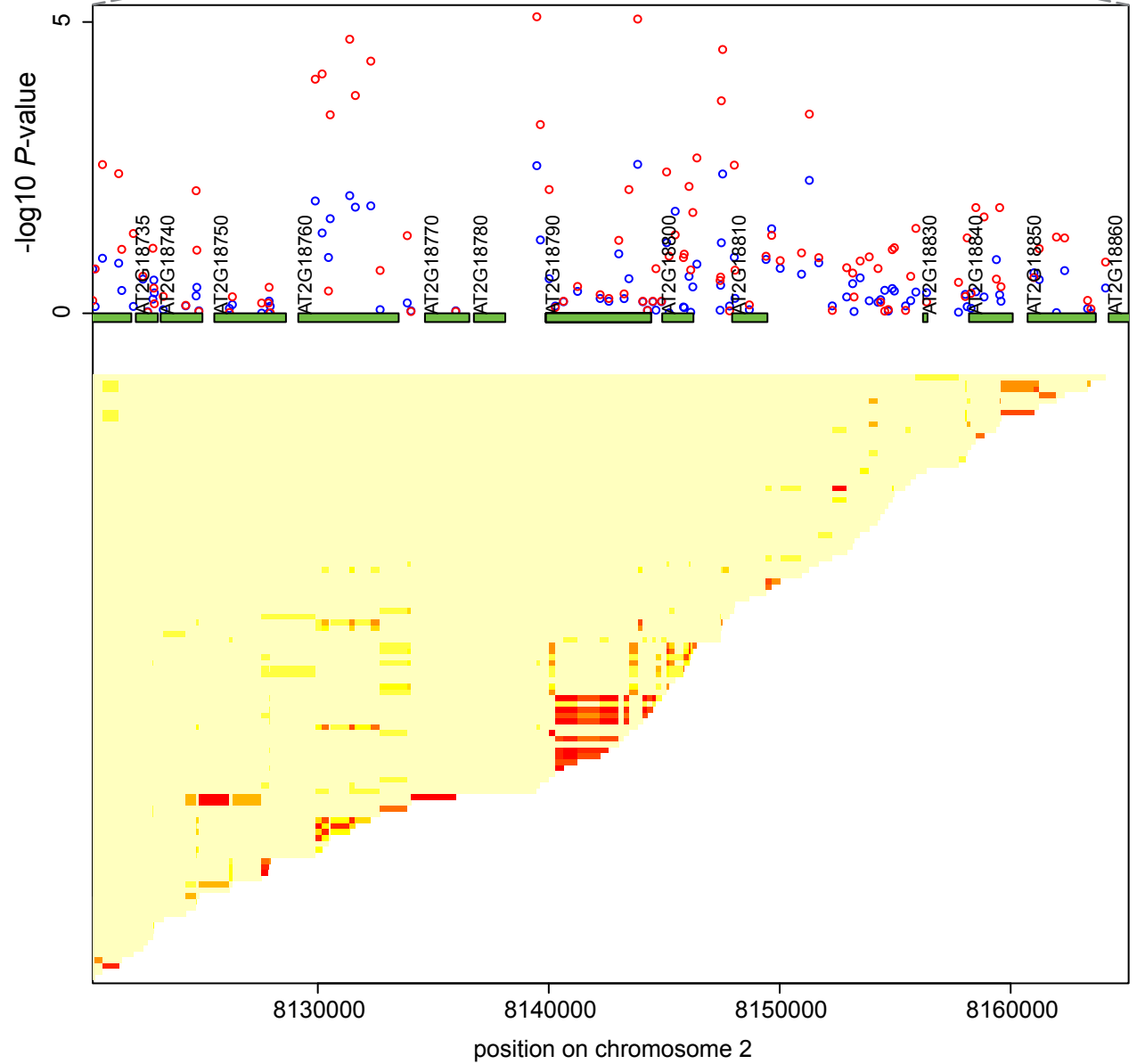

Supplement: Figure S6 — Detailed view of associations with high R∶FR around PHYB. The lower panel is a detailed view of the area highlighted by the green box in the upper panel. In both panels, open circles indicate the −log10 P-value of the SNPs in the region. Blue circles represent EMMA P-values while red circles represent Kruskal-Wallis P-values. Green rectangles running horizontally through the lower panel represent the genes +/−20 kb around PHYB. The pairwise linkage disequilibrium (R) between SNPs is indicated below the genes in the lower panel, with darker colors representing higher linkage disequilibrium. (PDF) [file pgen.1002589.s008.pdf]

# Kruskal Wallis

# EMMA

*a priori*

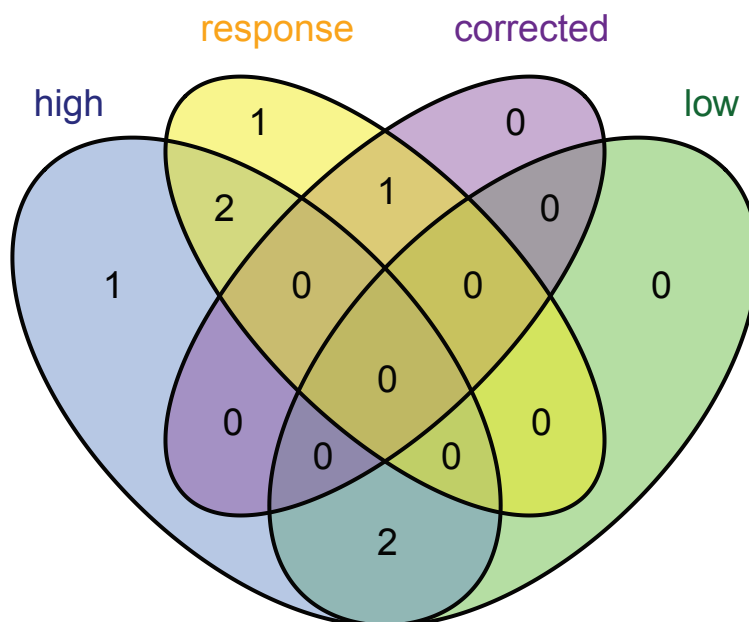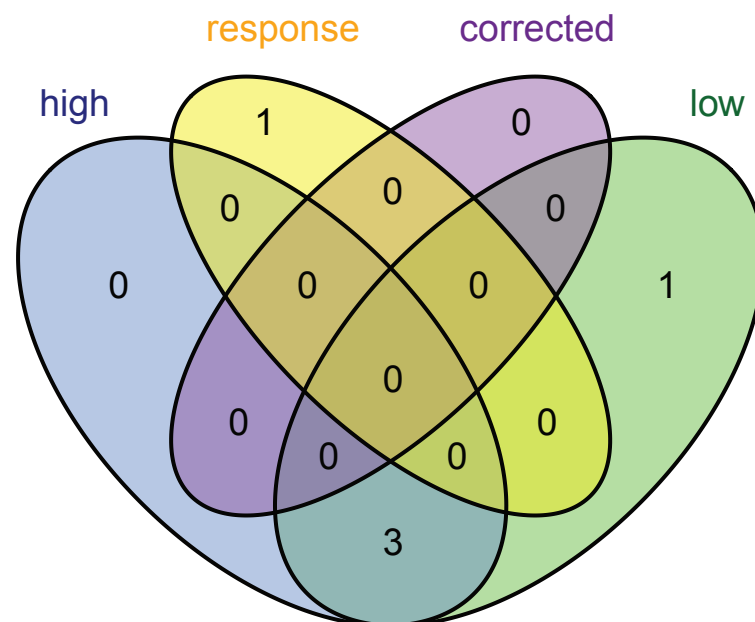

*all de novo*

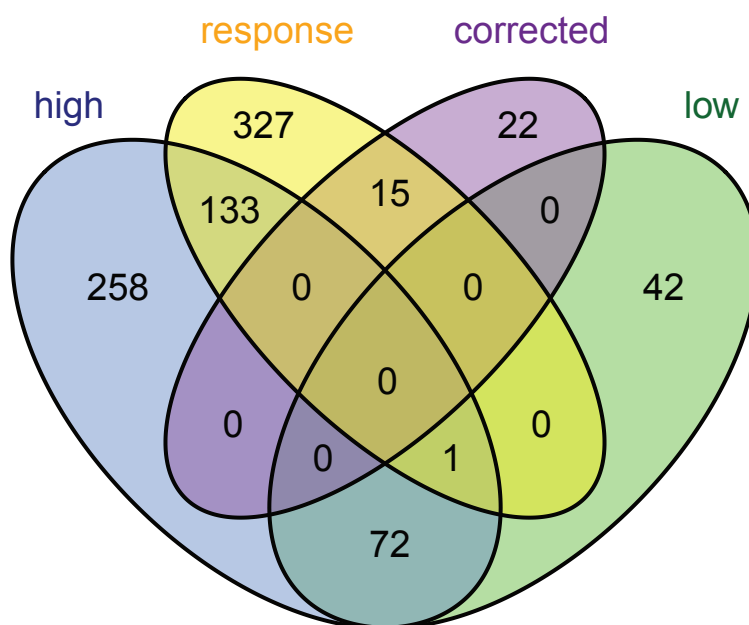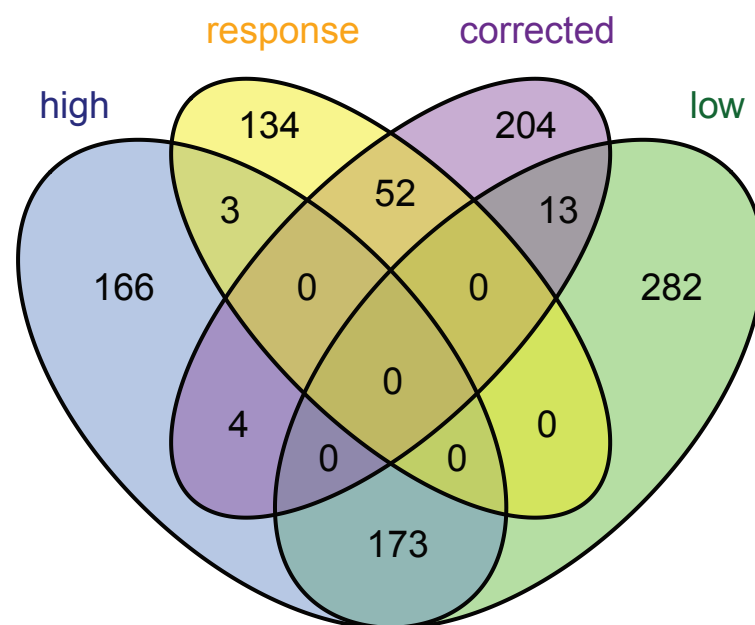

*selected de novo*

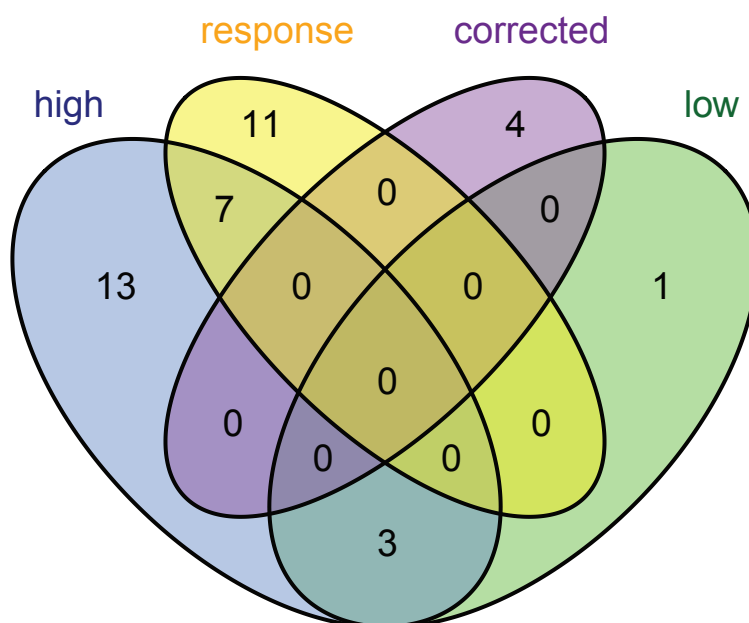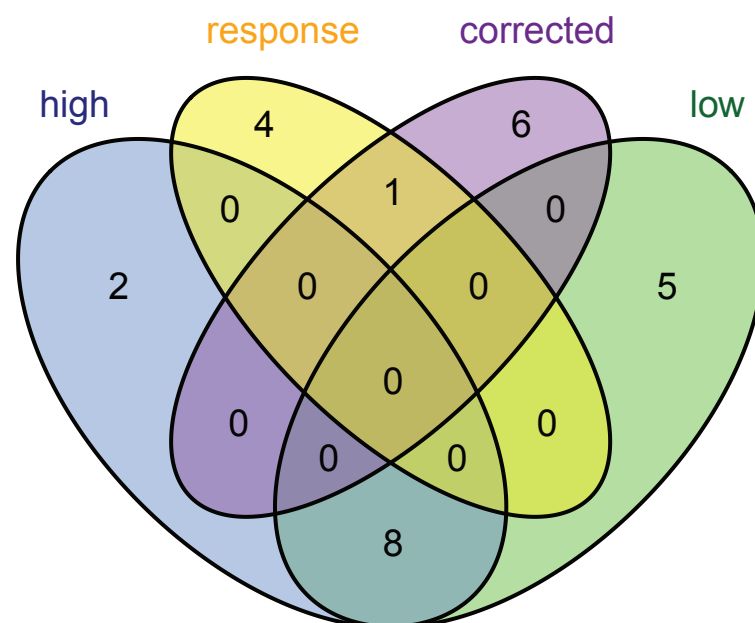

Supplement: Figure S7 — Venn diagrams of candidate gene lists. Venn diagrams showing the number of significant genes common to all combinations of the four study phenotypes. Diagrams for both Kruskal-Wallis and EMMA tests for the three candidate gene lists described in the text are presented. (PDF) [file pgen.1002589.s009.pdf]

**high R:FR**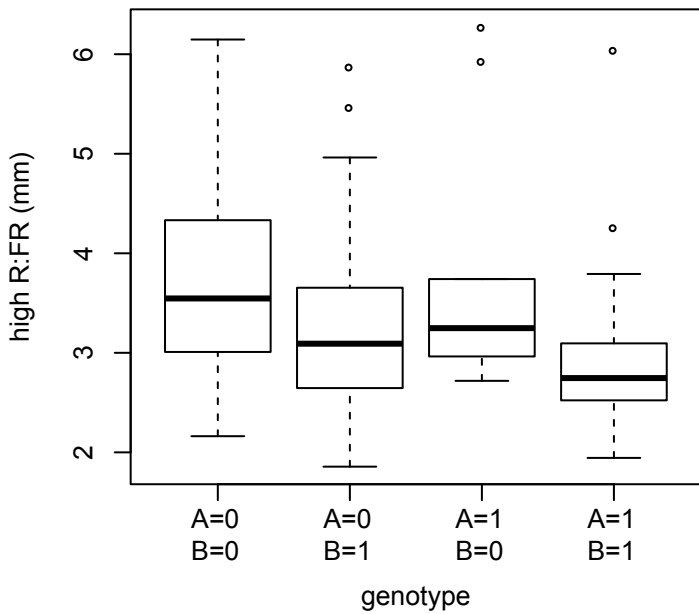**low R:FR**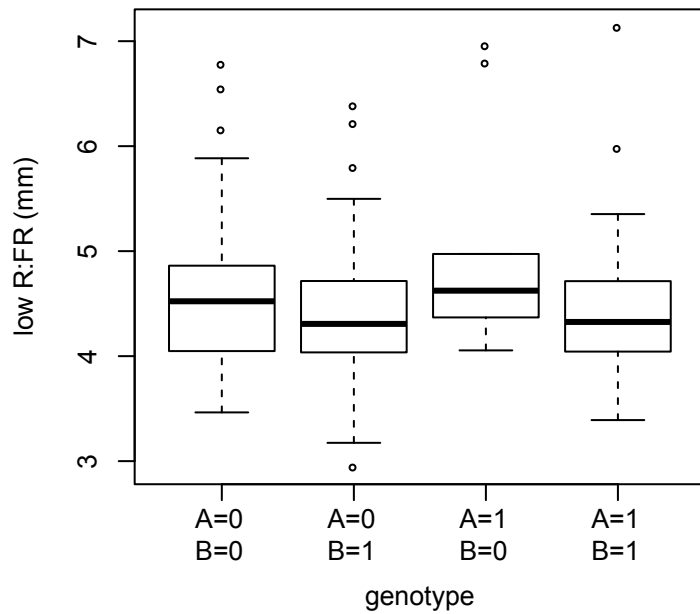**response to low R:FR**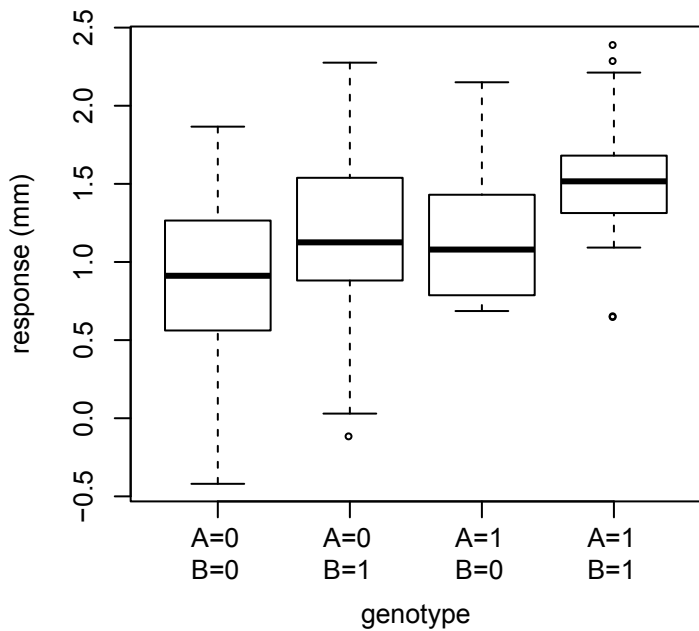**corrected response**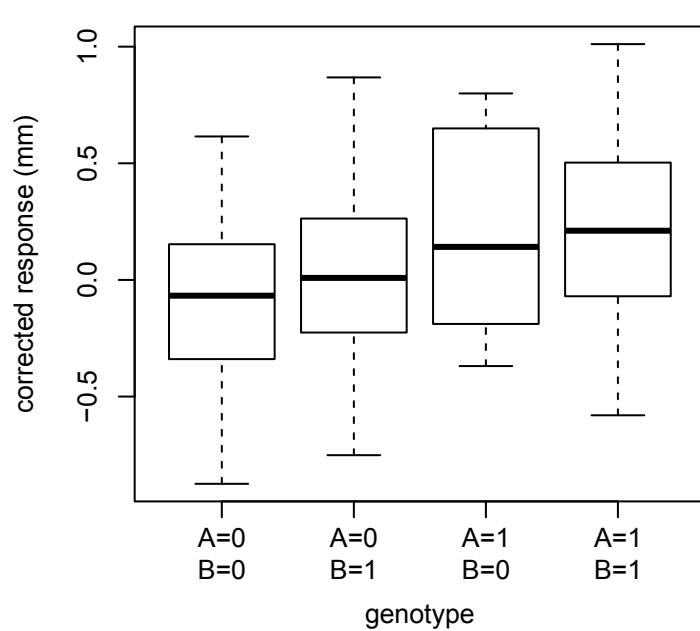

Supplement: Figure S8 — Phenotypes of accessions carrying the most significant SNPs around PHYA and PHYB. Box plots for all phenotypes. The four groups in each plot represent the four possible allelic combinations of the most significantly-associated SNPs around PHYA and PHYB. The PHYA SNP is Chr1:3079229 and the PHYB SNP is Chr2:8139482 (TAIR 9 annotation). The letter A in each genotype group designation denotes the PHYA genotype, while the letter B denotes the PHYB genotype. (PDF) [file pgen.1002589.s010.pdf]

**A**

high R:FR

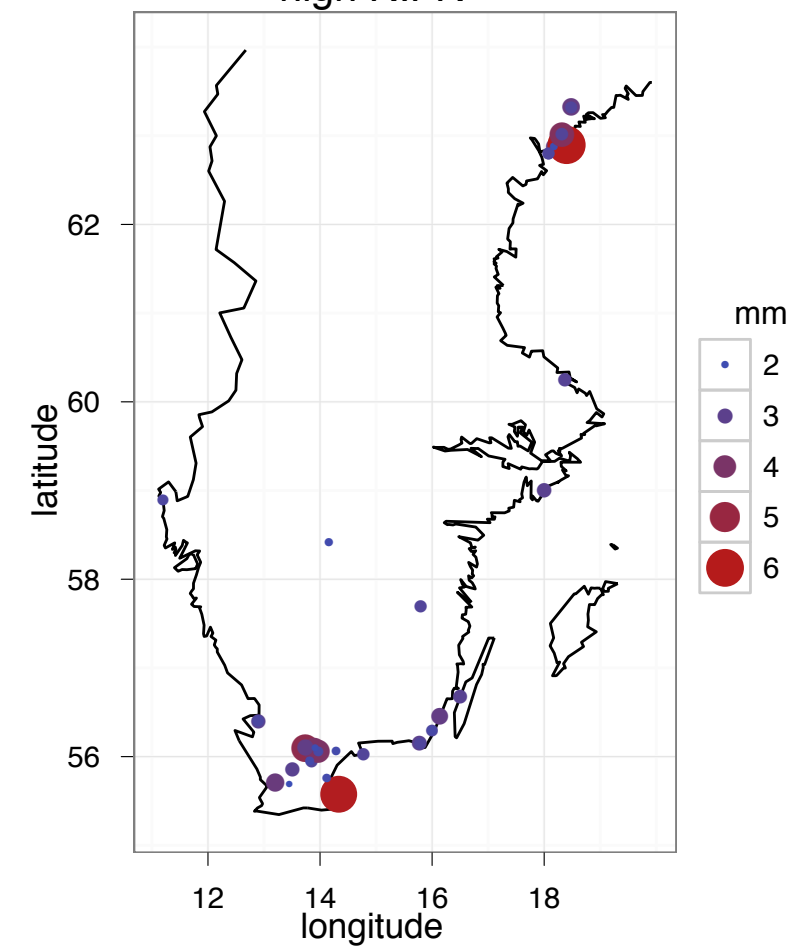**B**

low R:FR

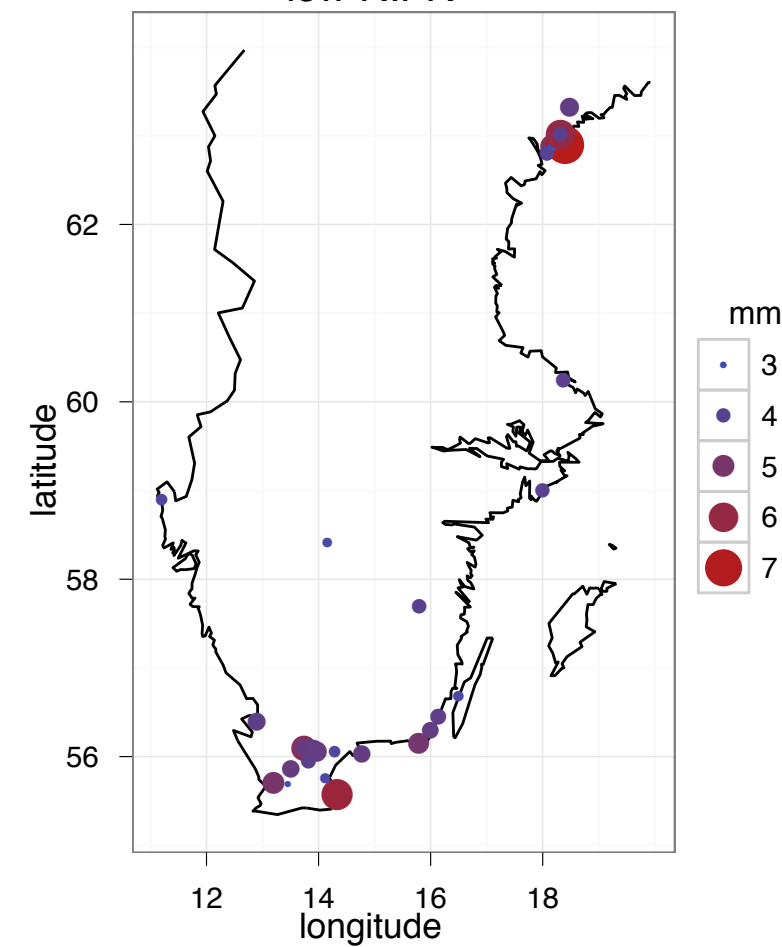**C**

response

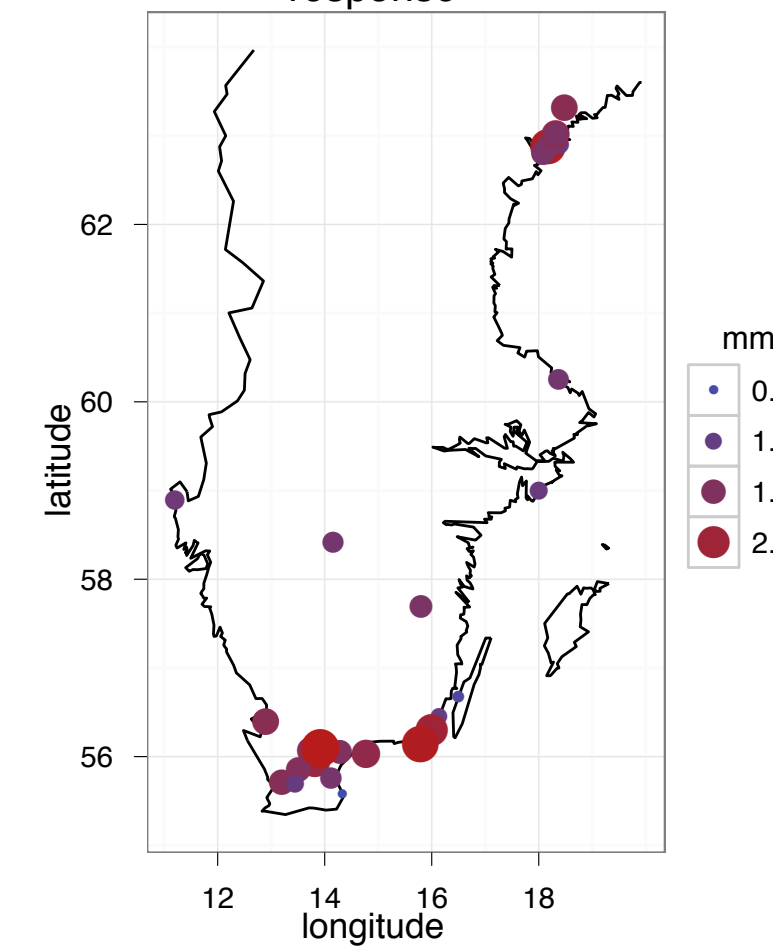**D**

corrected

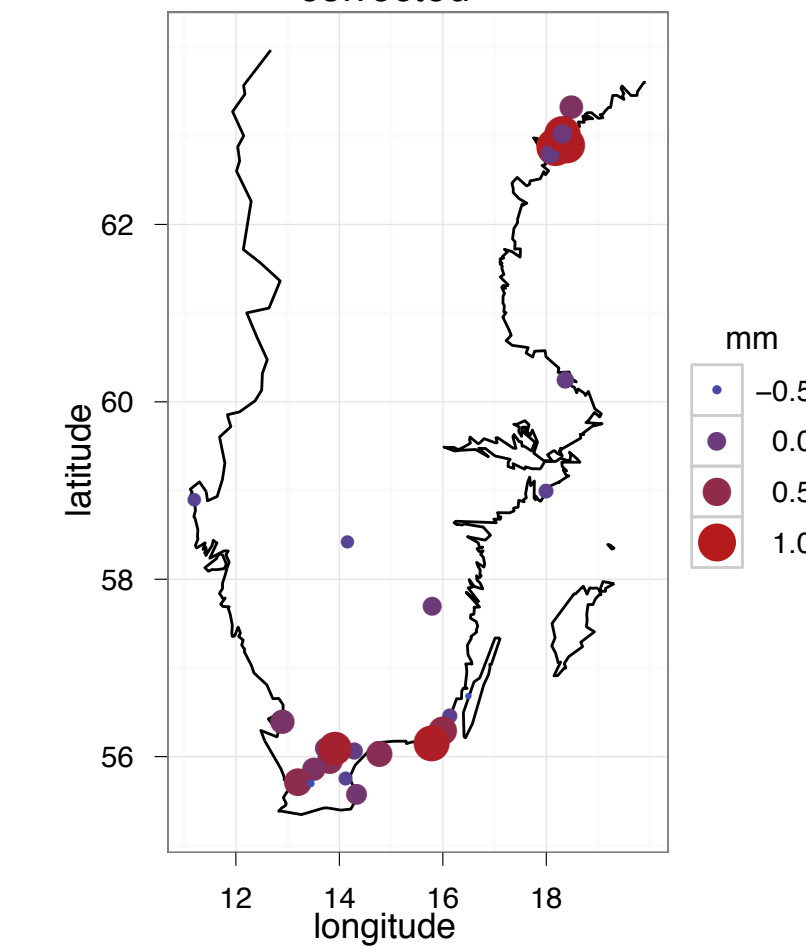**E**

PHYA

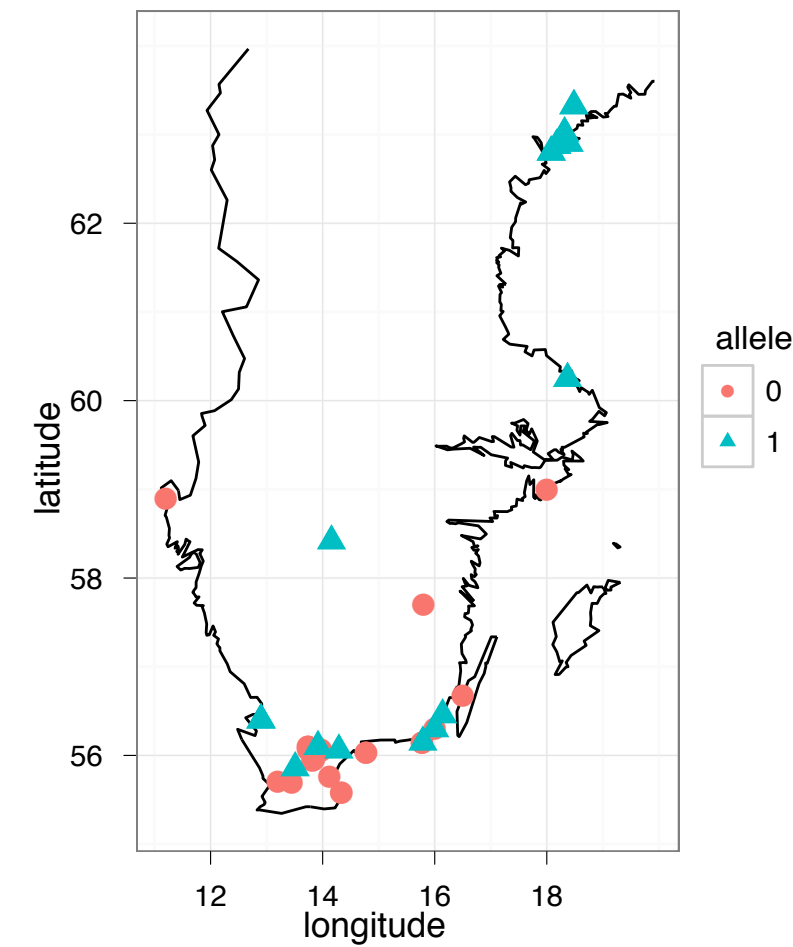**F**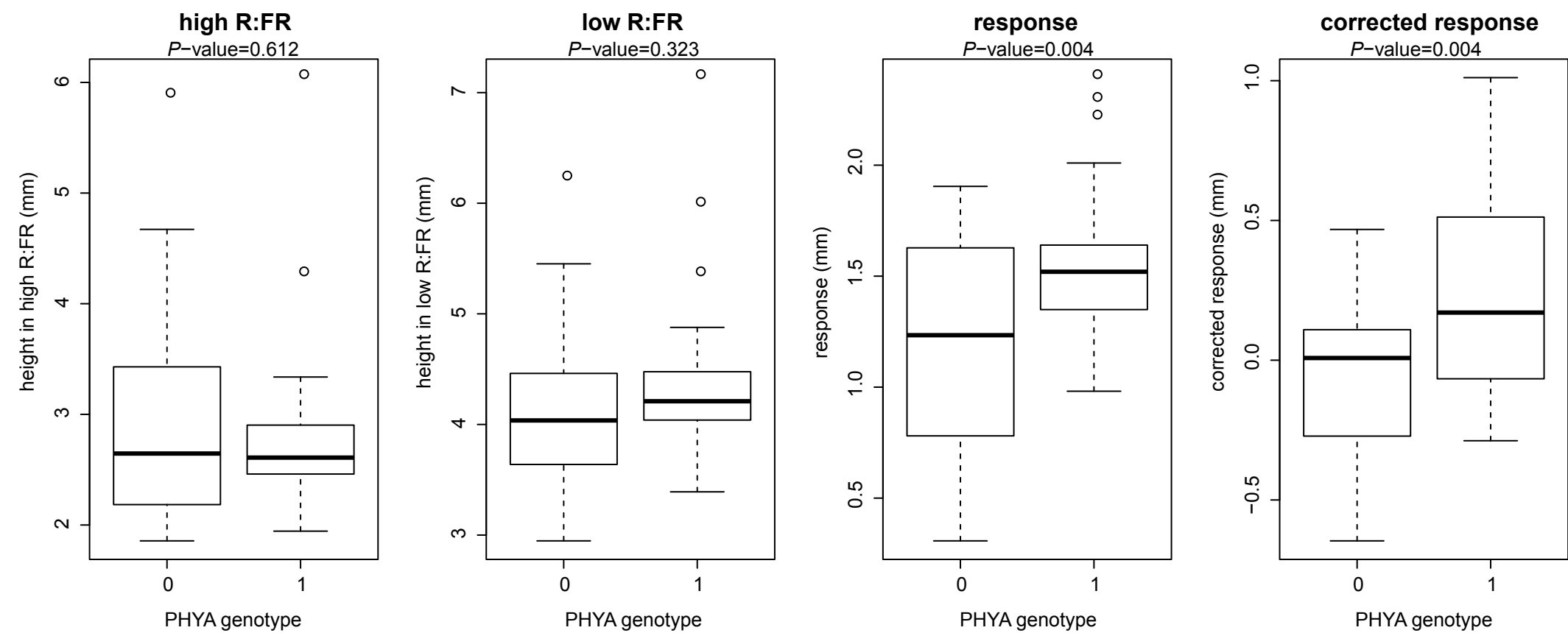

Supplement: Figure S9 — Phenotypes and PHYA/PIF3 variation in Swedish accessions. (A–D) Geographic distribution of phenotypic values. Phenotypic values are represented by a gradient in both size and color; small blue circles represent smaller values, while large red circles indicate larger values. (E) Geographic distribution of the alleles of the most significant SNP near PHYA/PIF3 (Chr1:3079229) for the Swedish accessions used in this study. (F) Box plots for all phenotypes grouped by the alleles represented in panel E. T-test P-values for differences in trait means between the alleles are presented above each box plot. (PDF) [file pgen.1002589.s011.pdf]
