# Supplementary material for: A Genome-Wide Association Study Identifies Variants Underlying the Arabidopsis thaliana Shade Avoidance Response
Source: PLoS Genet. 2012 Mar 15;8(3):e1002589. doi: 10.1371/journal.pgen.1002589 (PMC3305432; doi:10.1371/journal.pgen.1002589)
Supplement: Figure S2 — Correlations with the corrected response phenotype. Correlations between the corrected response phenotype and hypocotyl height in high R∶FR (A), height in low R∶FR (B), and response to low R∶FR (C). (PDF) [file pgen.1002589.s004.pdf]

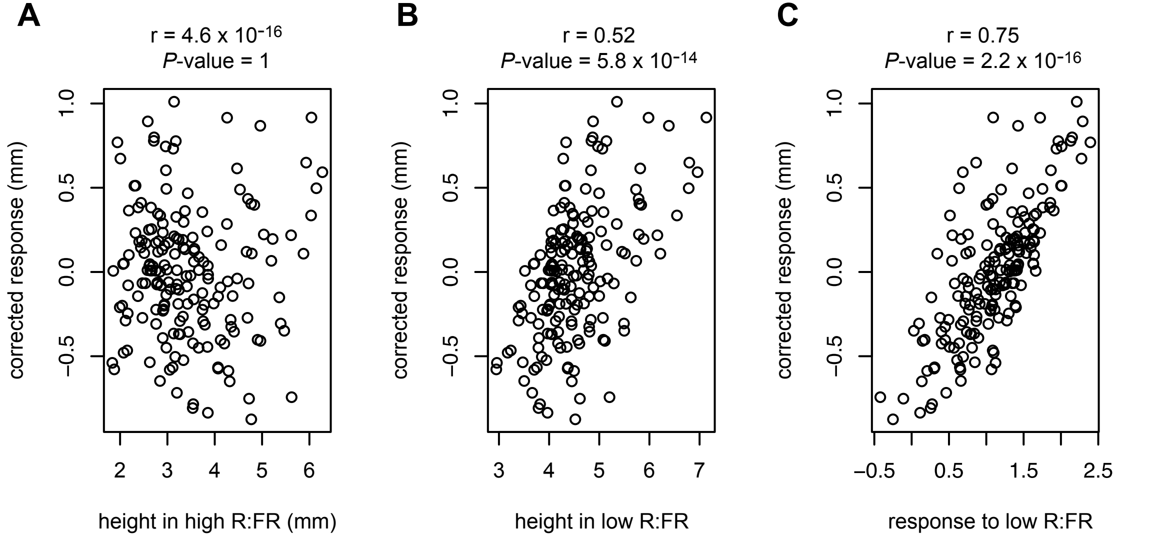
A
r = 4.6 x 10−16
P-value = 1
corrected response (mm)
height in high R:FR (mm)
B
r = 0.52
P-value = 5.8 x 10−14
corrected response (mm)
height in low R:FR
C
r = 0.75
P-value = 2.2 x 10−16
corrected response (mm)
response to low R:FR
