# Supplementary material for: A Genome-Wide Association Study Identifies Variants Underlying the Arabidopsis thaliana Shade Avoidance Response
Source: PLoS Genet. 2012 Mar 15;8(3):e1002589. doi: 10.1371/journal.pgen.1002589 (PMC3305432; doi:10.1371/journal.pgen.1002589)
Supplement: Table S1 — Arabidopsis thaliana accessions used in this study. (PDF) [file pgen.1002589.s012.pdf]

Supporting Table 1. *Arabdopsis thaliana* accessions used in the study.

Ecotype ID	Native Name	TAIR Stock Number	GWAS?
5837	Bor-1	CS22590	yes
6008	Duk	none	yes
6009	Eden-1	CS22572	yes
6016	Eds-1	none	yes
6039	Hovdala-2	none	yes
6042	Lom1-1	none	yes
6043	Lov-1	CS22574	yes
6046	Lov-5	CS22575	yes
6064	Nyl-2	none	yes
6074	Or-1	none	yes
6088	Stu1-1	none	no
6243	Tottarp-2	none	yes
6709	Bg-2	CS22342	yes
6897	Ag-0	CS22630	yes
6898	An-1	CS22626	yes
6899	Bay-0	CS22633	yes
6900	Bil-5	CS22578	yes
6901	Bil-7	CS22579	yes
6903	Bor-4	CS22591	yes
6905	Bur-0	CS22656	yes
6906	C24	CS22620	yes
6907	CIBC-17	CS22603	yes
6908	CIBC-5	CS22602	yes
6909	Col-0	CS22625	yes
6910	Ct-1	CS22639	yes
6911	Cvi-0	CS22614	yes
6913	Eden-2	CS22573	yes
6914	Edi-0	CS22657	yes
6915	Ei-2	CS22616	yes
6916	Est-1	CS22629	yes
6917	Fab-2	CS22576	yes
6918	Fab-4	CS22577	yes
6919	Ga-0	CS22634	yes
6920	Got-22	CS22609	yes
6921	Got-7	CS22608	yes
6922	Gu-0	CS22617	yes
6923	HR-10	CS22597	yes
6924	HR-5	CS22596	yes
6926	Kin-0	CS22654	yes
6927	Kno-10	CS22566	yes
6928	Kno-18	CS22567	yes
6929	Kondara	CS22651	yes
6930	Kz-1	CS22606	yes

6931	Kz-9	CS22607	yes
6932	Ler-1	CS22618	yes
6933	LL-0	CS22650	yes
6936	Lz-0	CS22615	yes
6937	Mrk-0	CS22635	yes
6938	Ms-0	CS22655	yes
6939	Mt-0	CS22642	yes
6940	Mz-0	CS22636	yes
6942	Nd-1	CS22619	yes
6943	NFA-10	CS22599	yes
6944	NFA-8	CS22598	yes
6945	Nok-3	CS22643	yes
6946	Oy-0	CS22658	yes
6951	Pu2-23	CS22593	yes
6956	Pu2-7	CS22592	yes
6957	Pu2-8	CS22449	yes
6958	Ra-0	CS22632	yes
6959	Ren-1	CS22610	yes
6960	Ren-11	CS22611	yes
6961	Se-0	CS22646	yes
6962	Sha	CS22652	yes
6963	Sorbo	CS22653	yes
6964	Spr1-2	CS22582	yes
6965	Spr1-6	CS22583	yes
6966	Sq-1	CS22600	yes
6967	Sq-8	CS22601	yes
6968	Tamm-2	CS22604	yes
6969	Tamm-27	CS22605	yes
6970	Ts-1	CS22647	yes
6971	Ts-5	CS22648	yes
6972	Tsu-1	CS22641	yes
6973	Ull2-3	CS22587	yes
6975	Uod-1	CS22612	yes
6976	Uod-7	CS22613	yes
6977	Van-0	CS22627	yes
6978	Wa-1	CS22644	yes
6979	Wei-0	CS22622	yes
6980	Ws-0	CS22623	yes
6981	Ws-2	CS22659	yes
6982	Wt-5	CS22637	yes
6983	Yo-0	CS22624	yes
6984	Zdr-1	CS22588	yes
6985	Zdr-6	CS22589	yes
7081	Co	CS3180	yes
7258	Nw-0	CS6811	yes
7323	Rubezhnoe-1	CS927	yes

7327	Sf-1	CS6855	no
7340	Sav-0	CS6856	yes
7514	RRS-7	CS22564	yes
7515	RRS-10	CS22565	yes
7516	Var2-1	CS22580	yes
7517	Var2-6	CS22581	yes
7518	Omo2-1	CS22584	yes
7519	Omo2-3	CS22585	yes
7520	Lp2-2	CS22594	yes
7521	Lp2-6	CS22595	yes
7522	Mr-0	CS22640	yes
7523	Pna-17	CS22570	yes
7524	Rmx-A02	CS22568	yes
7525	Rmx-A180	CS22569	yes
7526	Pna-10	CS22571	yes
8213	Pro-0	CS22649	yes
8214	Gy-0	CS22631	yes
8215	Fei-0	CS22645	yes
8222	Lis-2	none	yes
8230	Algutsrum	none	yes
8231	Bro1-6	none	yes
8233	Dem-4	none	yes
8235	Hod	none	yes
8236	HSm	none	yes
8238	Kent	none	yes
8239	Koln	CS6003	yes
8241	Liarum	none	yes
8242	Lillo-1	none	yes
8244	PHW-34	CS6034	no
8245	Seattle-0	CS6187	yes
8247	San-2	none	yes
8249	Vimmerby	none	yes
8250	Will	none	no
8252	Alc-0	CS1656	no
8254	Ang-0	CS949	yes
8256	Ba1-2	none	yes
8257	Bå3-3	none	no
8258	Ba4-1	none	yes
8259	Ba5-1	none	yes
8264	Bla-1	CS971	yes
8265	Blh-1	CS1031	no
8266	Boo2-1	none	yes
8270	Bs-1	CS997	yes
8271	Bu-0	CS1007	yes
8274	Can-0	CS1065	yes
8275	Cen-0	CS1067	yes

8283	Dra3-1	none	yes
8284	DraII-1	none	yes
8285	DraIII-1	none	yes
8290	En-1	CS1137	yes
8296	Gd-1	CS1185	yes
8297	Ge-0	CS1187	yes
8300	Gr-1	CS1199	yes
8303	H55	CS923	no
8304	Hi-0	CS1227	yes
8310	Hs-0	CS1237	yes
8311	In-0	CS1239	yes
8312	Is-0	CS1241	yes
8313	Jm-0	CS1259	yes
8314	Ka-0	CS1267	yes
8321	Kz-13	CS22445	no
8323	Lc-0	CS1307	yes
8325	Lip-0	CS1337	yes
8326	Lis-1	none	yes
8329	Lm-2	CS1345	yes
8334	Lu-1	CS1353	yes
8337	Mir-0	CS1379	yes
8343	Na-1	CS1385	yes
8351	Ost-0	CS1431	yes
8353	Pa-1	CS1439	yes
8354	Per-1	CS1445	yes
8355	Petergof	CS926	no
8356	Pi-0	CS1455	no
8357	Pla-0	CS1459	yes
8365	Rak-2	CS1485	yes
8369	Rev-1	none	yes
8374	Rsch-4	CS1494	yes
8377	Santa Clara	CS8069	no
8378	Sap-0	CS1507	yes
8386	Sr:5	none	yes
8387	St-0	CS1535	yes
8388	Stw-0	CS1539	yes
8389	Ta-0	CS1549	yes
8395	Tu-0	CS1567	yes
8420	Kelsterbach-4	CS6041	yes
8422	Fja1-1	none	yes
8423	Hov2-1	none	yes
8424	Kas-2	CS6751	yes
8426	Ull1-1	none	yes
9057	Vinslov	none	yes
100000	Wil-1-Dean-Lab	none	yes
