# Supplementary material for: A Genome-Wide Association Study Identifies Variants Underlying the Arabidopsis thaliana Shade Avoidance Response
Source: PLoS Genet. 2012 Mar 15;8(3):e1002589. doi: 10.1371/journal.pgen.1002589 (PMC3305432; doi:10.1371/journal.pgen.1002589)
Supplement: Table S2 — Number of SNPs consided significant for all cutoff criteria used in this study. (PDF) [file pgen.1002589.s013.pdf]

Supporting Table 2. Number of SNPs considered significant for all cutoff criteria used in this study.

			Number of SNPs considered significant			
**Method**	**Cutoff (-log10 P-value)**	**SNP set***	**High R:FR**	**Low R:FR**	**Response**	**Corrected response**
KW	4	unfiltered	500	152	533	86
KW	5	filtered	67	12	87	12
EMMA	4	unfiltered	53	66	40	68
EMMA	4	filtered	24	37	39	55

*unfiltered SNP sets were used for *a priori* analysis, while *de novo* analysis used SNP sets filtered for a minor allele frequency>0.1.
