# Supplementary material for: A Genome-Wide Association Study Identifies Variants Underlying the Arabidopsis thaliana Shade Avoidance Response
Source: PLoS Genet. 2012 Mar 15;8(3):e1002589. doi: 10.1371/journal.pgen.1002589 (PMC3305432; doi:10.1371/journal.pgen.1002589)
Supplement: Table S3 — Characterization of the significant SNPs identified in a priori Kruskal Wallis tests, including position, minor allele frequency, and P-value rank. (PDF) [file pgen.1002589.s014.pdf]

Supporting Table 3. Characterization of significant SNPs identified in *a priori* Kruskal Wallis tests.

phenotype	Locus	Chromosome	Position	MAF	*P*-value (-log10)	*P*-value rank
high	AT2G18790	2	8129890	0.43	4.02	485
		2	8130188	0.28	4.11	404
		2	8131381	0.30	4.70	134
		2	8132295	0.31	4.33	275
		2	8139482	0.50	5.09	60
		2	8143850	0.27	5.05	65
		2	8147530	0.25	4.53	195
	AT3G15540	3	5261708	0.14	7.05	2
		3	5262842	0.17	6.24	11
		3	5265393	0.15	6.12	13
		3	5269429	0.15	6.10	14
		3	5273280	0.15	5.21	50
	AT3G03450	3	823785	0.30	4.58	170.5
		3	825049	0.30	4.58	170.5
		3	825333	0.30	4.45	229
	AT4G32280	4	15569081	0.38	5.14	55
	AT5G51810	5	21053332	0.15	4.22	332
		5	21054108	0.37	4.20	344
low	AT3G15540	3	5261708	0.14	6.15	1
		3	5262842	0.17	4.99	13
		3	5265393	0.15	4.71	36
		3	5269429	0.15	4.83	26
		3	5273280	0.15	4.10	128
	AT5G51810	5	21053089	0.22	4.35	76
		5	21053332	0.15	4.55	53
		5	21054108	0.37	4.07	135
response	AT1G04180	1	1099222	0.49	4.18	384
	AT2G01570	2	272607	0.49	4.12	441
	AT2G18790	2	8139482	0.50	6.18	22
		2	8143850	0.27	4.12	442.5
	AT4G32280	4	15567909	0.21	4.27	337
		4	15569081	0.38	5.68	38
corrected	AT1G04180	1	1102314	0.25	4.06	73
