# Supplementary material for: A Genome-Wide Association Study Identifies Variants Underlying the Arabidopsis thaliana Shade Avoidance Response
Source: PLoS Genet. 2012 Mar 15;8(3):e1002589. doi: 10.1371/journal.pgen.1002589 (PMC3305432; doi:10.1371/journal.pgen.1002589)
Supplement: Table S4 — Characterization of the significant SNPs identified in a priori EMMA tests, including position, minor allele frequency, P-value rank, effect size, and EMMA variance components. (PDF) [file pgen.1002589.s015.pdf]

Supporting Table 4. Description of significant SNPs identified in *a priori* EMMA tests.

Phenotype	Locus	Chromosome	Position	MAF	*P*-value (-log10)	*P*-value rank	effect size (mm)	variance from EMMA	
								genetic	random
high	AT3G15540	3	5261708	0.14	4.84	7	1.30	0.261	1.654
		3	5262842	0.17	4.21	35	1.08	0.233	1.813
	AT4G16780	4	9446718	0.04	4.02	51	1.68	0.192	1.999
	AT4G25420	4	12995815	0.04	4.43	20	1.83	0.184	2.008
low	AT3G15540	3	5261708	0.14	4.41	29	1.30	0.261	1.654
	AT4G16780	4	9446718	0.04	4.89	11	1.68	0.192	1.999
	AT4G25420	4	12995815	0.04	4.33	31	1.83	0.184	2.008
	AT5G51810	5	21053332	0.15	4.47	26	1.11	0.176	2.089
response	AT5G43890	5	17634683	0.48	4.13	37	0.43	0.173	2.189
