# Supplementary material for: A Genome-Wide Association Study Identifies Variants Underlying the Arabidopsis thaliana Shade Avoidance Response
Source: PLoS Genet. 2012 Mar 15;8(3):e1002589. doi: 10.1371/journal.pgen.1002589 (PMC3305432; doi:10.1371/journal.pgen.1002589)
Supplement: Table S5 — GWAS results for loci selected as interesting de novo candidate genes. (PDF) [file pgen.1002589.s016.pdf]

Supporting Table 5. GWAS results for loci selected as interesting *de novo* candidate genes.

		Kruskal-Wallis -log10 *P*-value				EMMA -log10 *P*-value					
**Locus**	**Gene name**	**High R:FR**	**Low R:FR**	**Response**	**Corrected**	**High R:FR**	**Low R:FR**	**Response**	**Corrected**	**Differential regulation**	**Pattern category***
AT1G09530	*PIF3*	3.49	0.99	5.10	4.12	1.41	0.98	2.76	4.26	TRUE	3
AT1G09540	*MYB61*	3.49	0.99	5.10	4.12	1.41	0.98	2.76	4.26	FALSE	3
AT1G09570	*PHYA*	3.49	0.99	5.10	3.57	1.41	0.98	2.76	3.79	TRUE	3
AT1G10170	*NFXL1*	3.01	1.43	4.91	5.34	3.18	1.93	3.68	6.13	TRUE	3
AT1G10210	*MPK1*	3.01	2.59	4.91	5.34	3.18	1.59	3.68	6.13	FALSE	3
AT1G10240	*FRS11*	3.48	2.82	4.91	5.34	1.68	1.59	3.23	6.13	FALSE	3
AT1G15050	*IAA34*	2.72	2.61	5.31	3.14	0.90	1.98	2.82	3.25	FALSE	3
AT1G19350	*BES1*	6.07	5.77	3.87	1.87	2.63	2.55	1.51	1.17	FALSE	1
AT1G48410	*AGO1*	5.14	4.54	4.16	2.81	2.15	2.38	2.80	2.19	FALSE	1
AT1G69640	*SBH1*	2.21	2.10	1.51	1.55	3.93	4.86	1.66	3.93	TRUE	1
AT2G18790	*PHYB*	5.09	2.77	6.18	1.84	2.56	1.56	3.97	1.91	TRUE	2
AT2G26990	*FUS12*	2.59	3.54	1.25	1.99	3.58	4.50	1.25	1.58	FALSE	1
AT2G27050	*EIL1*	1.15	2.74	1.25	2.82	2.68	1.28	4.14	1.92	TRUE	3
AT2G28190	*CSD2*	3.49	1.61	5.07	3.50	1.36	1.54	2.61	2.73	TRUE	3
AT2G28200	none	2.39	1.61	5.07	3.50	0.99	1.54	1.77	2.73	TRUE	3
AT2G32250	*FRS2*	7.05	2.88	4.95	2.53	2.59	2.20	2.94	1.90	FALSE	1
AT2G32410	*AXL*	5.12	2.23	3.49	1.46	1.84	1.60	2.33	1.45	FALSE	1
AT2G32440	*KAO1*	5.12	2.23	3.98	2.29	1.84	1.32	2.33	1.03	FALSE	1
AT2G32460	*MYB101*	5.12	2.23	3.98	2.29	1.84	1.32	2.33	1.03	FALSE	1
AT2G35300	*LEA18*	2.47	2.75	2.67	2.35	4.14	4.60	1.00	1.97	TRUE	1
AT2G46530	*ARF11*	5.87	4.43	4.42	2.05	2.25	2.03	3.14	1.85	TRUE	1
AT3G01220	*ATHB20*	5.09	4.64	3.15	1.59	1.41	1.80	1.91	1.83	FALSE	1
AT3G05800	*AIF1*	1.97	1.75	2.43	3.06	2.85	5.19	2.05	3.62	TRUE	1
AT3G12580	*HSP70*	5.40	3.66	5.31	1.45	2.98	1.52	3.27	1.56	TRUE	2
AT3G12630	none	6.42	4.23	5.51	1.45	3.28	2.28	2.55	1.56	TRUE	2
AT3G12830	*SAP5*	4.19	5.14	3.68	2.60	2.23	2.32	2.12	2.15	FALSE	1
AT3G15540	*IAA19*	7.05	6.15	3.73	0.73	4.84	4.41	2.31	0.87	TRUE	1
AT3G15570	none	7.05	6.15	3.73	0.88	4.84	4.41	2.65	1.01	FALSE	1
AT3G23210	*bHLH34*	6.05	3.56	3.70	4.08	2.83	3.28	2.47	3.86	TRUE	1
AT3G25880	none	3.96	4.32	3.10	1.85	4.06	5.32	1.85	2.35	FALSE	1
AT3G25890	none	3.96	3.67	3.10	1.85	4.06	5.32	2.04	1.81	TRUE	1
AT3G59470	none	5.36	4.87	2.44	1.67	2.97	2.63	1.83	1.35	FALSE	1
AT3G59900	*ARGOS*	5.91	3.71	3.27	1.70	2.90	2.05	1.74	1.23	TRUE	1
AT4G01120	*GBF2*	5.51	1.71	6.31	3.97	1.80	1.31	3.53	3.87	FALSE	2
AT4G01350	none	4.58	2.21	5.69	4.03	2.02	1.43	3.55	3.86	FALSE	3
AT4G01480	*PPa5*	3.75	2.47	6.00	4.43	1.98	1.09	5.53	4.04	TRUE	3
AT4G08810	*SUB1*	4.60	4.89	2.71	1.57	4.33	3.08	2.34	1.07	FALSE	1
AT4G16780	*ATHB-2*	3.39	2.16	3.19	1.82	4.02	4.89	2.27	1.55	TRUE	1
AT4G19990	*FRS1*	3.21	3.45	1.94	2.19	4.22	3.87	2.01	1.74	FALSE	1
AT4G26150	*CGA1*	5.30	3.93	4.62	2.14	7.08	5.86	2.76	1.35	TRUE	1
AT4G26200	*ACS7*	5.30	3.93	4.62	2.47	7.08	5.86	2.76	1.35	TRUE	1
AT4G32280	*IAA29*	5.14	1.73	5.68	1.69	1.39	1.51	1.61	1.24	TRUE	2
AT4G32980	*ATH1*	5.21	1.11	7.43	3.08	3.61	1.31	4.93	2.66	FALSE	2
AT4G35230	*BSK1*	2.30	2.08	6.00	3.68	3.07	2.80	3.13	3.47	FALSE	3
AT5G01990	none	5.01	3.13	3.81	1.70	3.23	1.78	3.19	1.36	FALSE	1
AT5G35840	*PHYC*	4.83	3.23	4.57	3.64	2.02	1.81	4.18	3.42	FALSE	3
AT5G43700	*ATAUX2-11*	3.38	3.26	3.07	2.96	3.38	4.51	1.93	2.71	TRUE	1
AT5G43890	*YUC5*	3.38	2.40	3.79	2.89	2.61	1.57	4.13	2.59	TRUE	3
AT5G44110	*ABCI21*	1.38	2.52	3.78	5.41	1.70	1.91	2.86	4.70	FALSE	3
AT5G51810	*GA20OX2*	4.22	4.55	3.21	2.23	3.80	4.47	1.28	1.77	TRUE	1
AT5G57090	*EIR1*	5.46	1.57	6.05	2.53	3.91	2.11	3.26	1.70	FALSE	2
AT5G57360	*ZTL*	4.73	2.56	5.13	2.03	2.82	2.42	3.33	1.84	FALSE	3
AT5G63870	*PP7*	3.26	1.72	5.49	3.56	1.40	1.85	1.84	1.93	FALSE	3

*Significance pattern categories: 1=general control of hypocotyl height, 2=control of shade avoidance via hypocotyl height, 3=specific control of shade avoidance response.
