# Supplementary material for: A Genome-Wide Association Study Identifies Variants Underlying the Arabidopsis thaliana Shade Avoidance Response
Source: PLoS Genet. 2012 Mar 15;8(3):e1002589. doi: 10.1371/journal.pgen.1002589 (PMC3305432; doi:10.1371/journal.pgen.1002589)
Supplement: Table S6 — Difference in allelic means and t-test P-values for the most significant PHYA and PHYB SNPs. (PDF) [file pgen.1002589.s017.pdf]

Supporting Table 6. Difference in allelic means and *t*-test *P*-values for the most significant *PHYA* and *PHYB* SNPs.

	High R:FR		Low R:FR		Response		Corrected	
Locus	Difference (mm)	*P*-value	Difference (mm)	*P*-value	Difference (mm)	*P*-value	difference (mm)	*P*-value
PHYA	0.39	0.04	-0.05	0.73	-0.44	3.8E-06	-0.30	1.5E-04
PHYB	0.62	2.9E-05	0.24	0.04	-0.38	1.2E-06	-0.16	6.6E-03
