# Supplementary material for: A Genome-Wide Association Study Identifies Variants Underlying the Arabidopsis thaliana Shade Avoidance Response
Source: PLoS Genet. 2012 Mar 15;8(3):e1002589. doi: 10.1371/journal.pgen.1002589 (PMC3305432; doi:10.1371/journal.pgen.1002589)
Supplement: Table S7 — Phytochrome B and C genotyping results. (PDF) [file pgen.1002589.s018.pdf]

Supporting Table 7. Phytochrome B and C genotyping results

Accession	PHYC	PHYB Site 1	Site 3	Site 4	Site 7	Site 12
5837	Fr-2	Cvi	Cvi	Ler	Ler	Cvi
6008	Col	Cvi	Ler	Ler	Ler	Ler
6009	Fr-2	Ler	Ler	Ler	Ler	Ler
6016	Col	Ler	Ler	Ler	Ler	Ler
6039	Col	Cvi	Ler	Ler	Ler	Ler
6040	Fr-2	Cvi	Cvi	Ler	Ler	Cvi
6042	Fr-2	Cvi	Cvi	Ler	Ler	Cvi
6043	Fr-2	Ler	Ler	Ler	Ler	Ler
6046	NA	Ler	Ler	Ler	Ler	Ler
6064	Col	Cvi	Ler	Ler	Ler	Ler
6074	Col	Cvi	Ler	Ler	Ler	Cvi
6088	Col	Cvi	NA	Ler	Ler	Cvi
6243	Col	Cvi	Cvi	Ler	Ler	Cvi
6709	Fr-2	Cvi	Cvi	Ler	Ler	Cvi
6897	Fr-2	Cvi	Cvi	Ler	Ler	Cvi
6898	Fr-2	Ler	Ler	Ler	Ler	Ler
6899	Col	Cvi	Cvi	NA	Ler	Cvi
6900	Col	Cvi	Ler	Ler	Ler	Ler
6901	Col	Cvi	Ler	Ler	Ler	Ler
6903	Fr-2	Cvi	Ler	Ler	Ler	Ler
6904	Fr-2	Cvi	Cvi	Ler	Ler	Cvi
6905	Fr-2	Cvi	Cvi	NA	Cvi	Cvi
6906	Fr-2	Cvi	Cvi	Ler	Ler	Cvi
6907	Fr-2	NA	NA	Ler	NA	Cvi
6908	Fr-2	Cvi	Cvi	Ler	Ler	Cvi
6909	Col	Ler	Ler	NA	Ler	Ler
6910	Fr-2	Cvi	Ler	Ler	Ler	Cvi
6911	NA	Cvi	Cvi	NA	Cvi	Cvi
6913	Fr-2	Ler	Ler	Ler	Ler	Ler
6914	Fr-2	Cvi	Cvi	NA	Ler	Cvi
6915	Fr-2	Cvi	Cvi	Ler	Ler	Cvi
6916	Ler	Cvi	Cvi	Ler	Ler	Cvi
6917	NA	Ler	Ler	Ler	Ler	Ler
6918	NA	NA	Cvi	NA	Cvi	Cvi
6919	Col	Cvi	Ler	Ler	Ler	Ler
6920	Fr-2	Cvi	Cvi	NA	Ler	Cvi
6921	Fr-2	Cvi	Cvi	Ler	Ler	Cvi
6922	Fr-2	Cvi	Cvi	NA	Ler	Cvi
6923	Fr-2	Cvi	Cvi	Ler	Ler	Cvi
6924	Fr-2	Cvi	Cvi	Ler	Ler	Cvi
6926	Fr-2	Cvi	Cvi	NA	Ler	Cvi
6927	Fr-2	Cvi	Cvi	Ler	Ler	Cvi
6928	Fr-2	Cvi	Cvi	Ler	Ler	Cvi

6929	NA	Cvi	Cvi	Ler	Ler	Cvi
6930	Fr-2	Cvi	Cvi	Cvi	Cvi	Cvi
6931	Col	Cvi	Cvi	Cvi	Cvi	Cvi
6932	Ler	Ler	Ler	Ler	Ler	Ler
6933	Fr-2	Cvi	Cvi	Cvi	Cvi	Cvi
6936	Fr-2	Cvi	Cvi	Ler	Ler	Cvi
6937	NA	Cvi	Cvi	Ler	Ler	Cvi
6938	Col	Cvi	Cvi	NA	Cvi	Cvi
6939	Col	Cvi	Cvi	Ler	Ler	Cvi
6940	NA	Cvi	Cvi	Ler	Cvi	Cvi
6942	Col	Cvi	Cvi	Ler	Ler	Cvi
6943	NA	Cvi	Cvi	Ler	Ler	Cvi
6944	Fr-2	Cvi	Cvi	Ler	Ler	Cvi
6945	Fr-2	Cvi	Cvi	Cvi	Cvi	Cvi
6951	Fr-2	Cvi	Cvi	NA	Cvi	Cvi
6956	Fr-2	Cvi	Cvi	Ler	Ler	Cvi
6957	NA	NA	NA	Ler	NA	NA
6958	Fr-2	Cvi	Cvi	NA	Cvi	Cvi
6959	Fr-2	Cvi	Cvi	Ler	Ler	Cvi
6960	Fr-2	Cvi	Cvi	Ler	Ler	Cvi
6961	Fr-2	Cvi	Cvi	Cvi	Cvi	Cvi
6962	NA	Cvi	Cvi	Ler	Ler	Cvi
6963	NA	Cvi	Cvi	Ler	Ler	Cvi
6964	Col	Cvi	Cvi	NA	Cvi	Cvi
6965	Col	Cvi	Cvi	Ler	Ler	Cvi
6966	Fr-2	Cvi	Cvi	Ler	Ler	Cvi
6967	Ler	Cvi	Cvi	NA	Ler	Cvi
6968	Col	NA	Cvi	Ler	Ler	Cvi
6969	Col	Cvi	Cvi	Ler	Ler	Cvi
6970	Fr-2	Cvi	Cvi	Cvi	Cvi	Cvi
6971	Fr-2	Cvi	Cvi	NA	Ler	Cvi
6972	Fr-2	Cvi	Cvi	NA	Ler	Cvi
6973	Ler	Cvi	Cvi	Ler	Ler	Cvi
6974	NA	Cvi	Cvi	Ler	Ler	Cvi
6975	Fr-2	Cvi	Cvi	Ler	Ler	Cvi
6976	Fr-2	Cvi	Ler	Ler	Ler	Ler
6977	Col	Cvi	Cvi	Ler	Ler	NA
6978	Col	Cvi	Cvi	Cvi	Cvi	Cvi
6979	NA	Cvi	Cvi	Ler	Ler	Cvi
6980	Col	Cvi	Ler	Ler	Ler	Cvi
6982	Ler	Cvi	Ler	Ler	Ler	Ler
6983	Fr-2	Cvi	Cvi	NA	Ler	Cvi
6984	Col	Cvi	Cvi	Ler	Ler	Cvi
6985	Col	Cvi	Ler	Ler	Ler	Ler
7033	Fr-2	Cvi	Cvi	Ler	Ler	Cvi
7081	Fr-2	Cvi	Cvi	Cvi	Cvi	Cvi

7258	Fr-2	Cvi	Cvi	Ler	Ler	Cvi
7323	Col	Cvi	Cvi	Ler	Ler	Cvi
7327	Fr-2	Cvi	Cvi	Cvi	Cvi	Cvi
7340	Col	Cvi	Ler	Ler	Ler	Ler
7438	Fr-2	Cvi	Cvi	Ler	Ler	Cvi
7514	Fr-2	Cvi	Cvi	Ler	Ler	Cvi
7515	Fr-2	Cvi	Cvi	Ler	Ler	Cvi
7516	Fr-2	Cvi	Cvi	Cvi	Cvi	Cvi
7517	Fr-2	Cvi	Ler	Ler	Ler	Ler
7518	Col	Cvi	Cvi	Ler	Ler	Cvi
7519	Col	Cvi	Cvi	Ler	Ler	Cvi
7520	Fr-2	Cvi	Ler	Ler	Ler	Ler
7521	Fr-2	Cvi	Cvi	Ler	Ler	Cvi
7522	Col	Cvi	Cvi	NA	Ler	Cvi
7523	Fr-2	Cvi	NA	Ler	Ler	Cvi
7524	Fr-2	Cvi	Cvi	NA	Ler	Cvi
7525	Fr-2	Cvi	Cvi	NA	Ler	Cvi
7526	Fr-2	Cvi	NA	Ler	Ler	Cvi
8213	Fr-2	Cvi	Cvi	NA	Ler	Cvi
8214	Fr-2	Cvi	Cvi	Ler	Ler	Cvi
8215	Fr-2	Cvi	Cvi	Cvi	Cvi	Cvi
8222	Col	Cvi	Cvi	Ler	Ler	Cvi
8230	Col	Cvi	Ler	Ler	Ler	Ler
8231	Col	Cvi	Ler	Ler	NA	Ler
8233	Fr-2	Cvi	Cvi	Ler	Ler	Cvi
8234	NA	NA	Ler	Ler	NA	NA
8235	Col	Ler	Ler	Ler	Ler	Ler
8236	Fr-2	Ler	Ler	Ler	Ler	Ler
8237	Col	Cvi	Cvi	Ler	Ler	Cvi
8238	Col	Cvi	Cvi	Cvi	Cvi	Cvi
8239	Ler	Cvi	Cvi	Cvi	Cvi	Cvi
8241	Col	Cvi	Cvi	Ler	Ler	Cvi
8242	Col	Cvi	Cvi	Ler	Ler	Cvi
8243	Fr-2	Cvi	Cvi	Ler	Ler	Cvi
8244	NA	Cvi	Cvi	Ler	Ler	Cvi
8245	Fr-2	Cvi	Cvi	Ler	Ler	Cvi
8246	Fr-2	Cvi	Cvi	Cvi	Cvi	Cvi
8247	Col	Cvi	Cvi	Ler	Ler	Cvi
8249	NA	Cvi	Ler	Ler	Ler	Ler
8250	Col	Cvi	Cvi	Cvi	Cvi	Cvi
8252	Fr-2	Cvi	Cvi	Cvi	Cvi	Cvi
8254	Fr-2	Cvi	Cvi	Ler	Ler	Cvi
8256	Col	Cvi	Cvi	Ler	Ler	Cvi
8257	Col	Cvi	Cvi	Ler	Ler	Cvi
8258	Ler	Cvi	Cvi	Ler	Ler	Cvi
8259	Ler	Cvi	Cvi	Ler	Ler	Cvi

8264	Fr-2	Cvi	Cvi	Cvi	Cvi	Cvi
8265	Ler	Cvi	Cvi	Ler	Ler	Cvi
8266	Col	Cvi	Ler	Ler	Ler	Ler
8270	Fr-2	Cvi	Cvi	Ler	Ler	Cvi
8271	Col	Cvi	Cvi	Ler	Ler	Cvi
8274	Fr-2	Cvi	Cvi	Ler	Ler	Cvi
8275	Col	Cvi	Cvi	Ler	Ler	Cvi
8283	Col	NA	Cvi	NA	Cvi	Cvi
8284	Fr-2	Cvi	Ler	Ler	Ler	Ler
8285	Col	Cvi	Ler	Ler	Ler	Ler
8290	Fr-2	NA	Ler	Ler	Ler	Cvi
8296	Ler	Ler	Ler	Ler	Ler	Ler
8297	Col	Cvi	Cvi	Cvi	Cvi	Cvi
8300	Fr-2	Cvi	Cvi	Ler	Ler	Cvi
8303	Col	Ler	Ler	Ler	Ler	Ler
8304	Fr-2	NA	Cvi	Ler	Ler	Cvi
8306	Col	Cvi	Cvi	Cvi	Cvi	Cvi
8310	NA	NA	NA	Ler	NA	Cvi
8311	Fr-2	Cvi	Cvi	Ler	Ler	Cvi
8312	Ler	Cvi	Cvi	Cvi	Cvi	Cvi
8313	Fr-2	Cvi	Cvi	Ler	Ler	Cvi
8314	Col	Cvi	Cvi	Ler	Ler	Cvi
8321	Col	Ler	Cvi	Cvi	Cvi	Cvi
8323	Col	Cvi	Cvi	Ler	Ler	Cvi
8325	NA	NA	Ler	Ler	NA	NA
8326	Col	Cvi	Ler	Ler	Ler	Cvi
8329	Fr-2	Cvi	Cvi	Ler	Ler	Cvi
8334	Col	Cvi	Ler	Ler	Ler	Cvi
8335	NA	Cvi	Cvi	Ler	Ler	Cvi
8337	NA	Cvi	Cvi	NA	Ler	Cvi
8343	Fr-2	Cvi	Cvi	Ler	Ler	Cvi
8351	Col	Cvi	Cvi	Ler	Ler	Cvi
8353	Fr-2	Cvi	Cvi	Ler	Ler	Cvi
8354	Col	Cvi	Cvi	Cvi	Cvi	Cvi
8355	Col	Cvi	Ler	Ler	Ler	Ler
8356	Fr-2	Cvi	Ler	Ler	Ler	Cvi
8357	Fr-2	Cvi	Cvi	Cvi	Cvi	Cvi
8365	Fr-2	Ler	Ler	Ler	Ler	Ler
8369	Fr-2	Cvi	Cvi	Ler	Ler	Cvi
8374	Col	Cvi	Cvi	Cvi	Cvi	NA
8376	Fr-2	Cvi	Ler	Ler	Ler	Ler
8377	Col	Ler	Ler	Ler	Ler	Ler
8378	Col	Cvi	Cvi	Ler	Ler	Cvi
8386	Fr-2	Cvi	Cvi	Cvi	Cvi	Cvi
8387	NA	NA	NA	Ler	NA	NA
8388	Col	Cvi	Cvi	Ler	NA	Cvi

8389	Col	Cvi	Cvi	Ler	Ler	Cvi
8395	Fr-2	Cvi	Cvi	Ler	Ler	Cvi
8420	NA	NA	NA	NA	NA	NA
8422	Fr-2	Cvi	Cvi	Ler	Ler	Cvi
8423	Fr-2	Cvi	Cvi	Cvi	Cvi	Cvi
8424	Fr-2	Cvi	Cvi	Ler	Ler	Cvi
8426	Col	NA	Cvi	Cvi	Cvi	Cvi
9057	NA	NA	NA	Ler	NA	NA
9058	Fr-2	Cvi	NA	Ler	NA	Ler
100000	NA	NA	NA	Cvi	NA	NA
